# Microporous Polymer Membranes: Molecular Stents Enhanced Solvent‐Accessibility for Organic Solvent Transport

**DOI:** 10.1002/advs.202416748

**Published:** 2025-05-29

**Authors:** Shuang Guo, Chuanjie Fang, Jiaqi Li, Xiaohe Wang, Weilin Feng, Hukang Guo, Ming Xie, Yongbing Zhuang, Young Moo Lee, Liping Zhu

**Affiliations:** ^1^ MOE Key Laboratory of Macromolecular Synthesis and Functionalization MOE Engineering Research Center of Membrane and Water Treatment Technology Department of Polymer Science and Engineering Zhejiang University Hangzhou 310027 China; ^2^ Department of Energy Engineering College of Engineering Hanyang University Seoul 04763 South Korea; ^3^ Department of Polymer Science and Engineering Department of Chemical Engineering University of Bath Bath BA2 7AY UK; ^4^ State Key Laboratory of Biochemical Engineering Institute of Process Engineering University of Chinese Academy of Sciences Chinese Academy of Sciences Beijing 100190 China

**Keywords:** free volume, microporous polymer membranes, molecular stents, organic solvent transport

## Abstract

Microporous polymer membranes with high solvent permeability are pivotal for upgrading molecular separations in organic solvents, but this remains challenging due to numerous sub‐0.4 nm ultra‐micropores resulting from local tight packing, which limit solvent‐accessibility. Herein, a microporous polyimide with high intrinsic free volume [PI‐TB‐NDI, naphthalenediimide (NDI) and Tröger's base (TB)] is synthesized for organic solvent nanofiltration. The resulting polymer showed high free volume because of fused aromatic rings and a twisted structure. Aromatic rings enhanced solvent resistance due to strong molecular interaction, but increased detrimental local tight packing as well. To suppress local tight packing without compromising the molecular interactions vital for stability, an *ortho*‐methyl group is deliberately introduced onto the TB unit to increase both intra‐ and inter‐molecular steric hindrance, imparting an H‐shaped TB‐NDI‐TB molecular stent. On the introduction of *ortho*‐methyl groups, the sub‐0.4 nm ultra‐micropores are enlarged to ultra‐micropores (0.6–0.7 nm) to give the membrane with rich solvent‐accessible sub‐nanochannels. This resulted in an unprecedented enhancement of solvent permeability, with ethanol permeability 2‐8 times greater than that of state‐of‐the‐art polymer membranes with similar selectivity. These findings advance the design strategy of microporous membranes with well‐tailored free volume without post‐treatments, enabling upscaling and efficient separation of precious species in organic solvents.

## Graphical abstract

A novel microporous polyimide (PI‐TB‐NDI) is designed for enhanced organic solvent nanofiltration. By incorporating *o*‐methyl groups into the Tröger's base (TB) unit to create molecular stents, the polymer's intrinsic free volume is effectively increased without the need for post‐treatment. This innovative approach transformed sub‐0.4 nm ultra‐micropores into ultra‐micropores (0.6–0.7 nm), significantly improving solvent‐accessibility for efficient molecular separations.

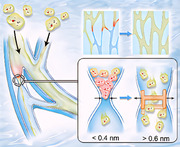

## 1 Introduction

The separation of commodity products from organic solvents is prevalent and significant in food, pharmaceutical, and chemical industries. However, it is a highly energy‐intensive process with up to 10%–15% of the world's total energy consumption, mainly because current separation processes are based on energy‐consuming evaporation, rectification, and distillation that rely on phase change.^[^ ^1^ ^]^ Membrane‐based organic solvent nanofiltration (OSN) technology, also known as solvent‐resistant nanofiltration, is a promising alternative due to its pure physical‐filtration feature without phase changes, endowing it with the high energy efficiency of up to one order of magnitude lower energy consumption than the current methods.^[^ ^2^ ^]^ However, traditional polymer membranes, such as cellulose acetate^[^ ^3^ ^]^ and polyimide^[^ ^4^ ^]^ membranes, usually show low solvent permeance due to their tight packing of molecular chains that lack permanent micropores. In addition, the selectivity of conventional polymer membranes is difficult to finely tailor due to their swelling in organic solvents, which changes the magnitude of micropores.^[^ ^5^ ^]^ Thus, their application remains a major challenge with a trade‐off between permeability and selectivity, and the solvent resistance of membranes.^[^ ^6^ ^]^

**Figure 1:**
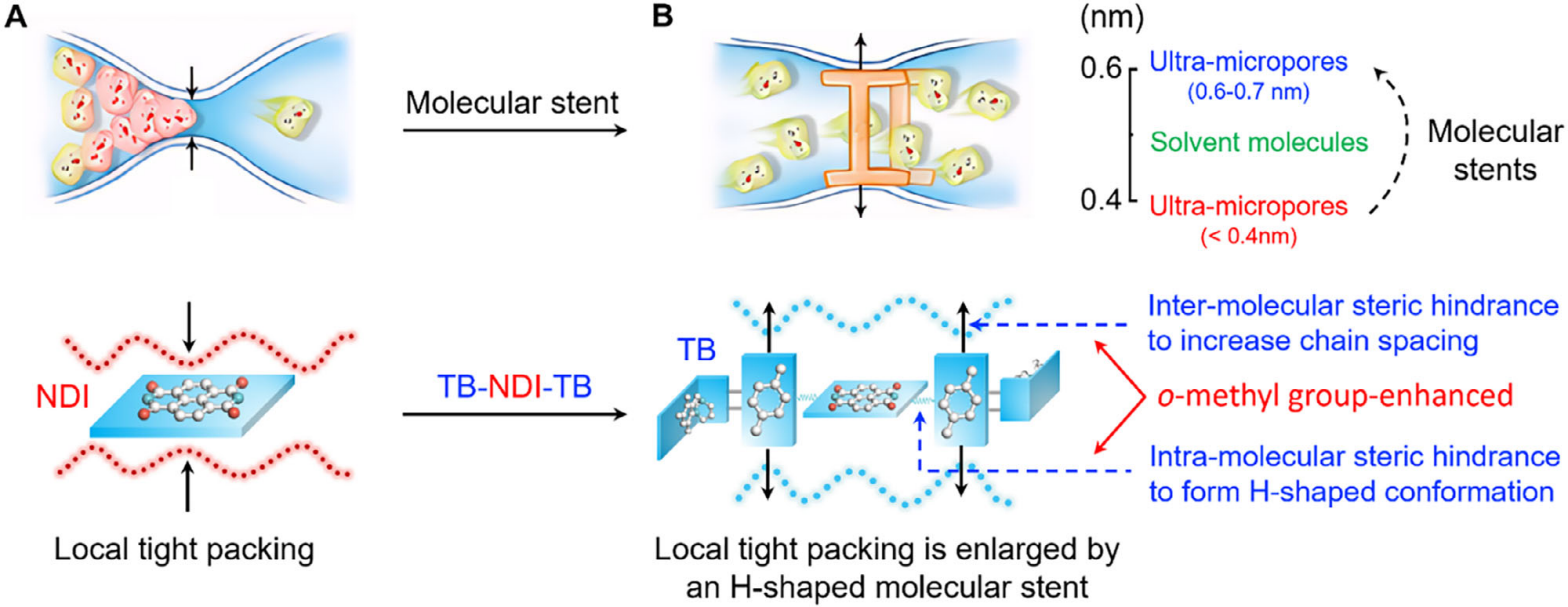
Concept of molecular stents and relationship between local tight packing, microporous structure, and solvent permeation. A) Local tight packing in microporous polymers forms sub‐0.4 nm ultra‐micropores, which are undesirable for transport of solvent clusters, akin to a locally narrowed or blocked artery to impede blood flow. B) Local tight packing is suppressed using the PI‐TB‐NDI molecular stents, which are formed by introducing *o*‐methyl groups onto the phenyl ring in the TB unit and structured in an H‐shape, creating ultra‐micropores of 0.6–0.7 nm, thereby enhancing solvent‐accessibility and facilitating efficient transport of solvent molecules.

Polymers of intrinsic microporosity (PIMs) are a class of microporous polymers.^[^ ^7^ ^]^ Fritsch^[^ ^8^ ^]^ and Livingston,^[^ ^9^ ^]^ and others first fabricated them as thin film composite membranes to show 40–90 times higher heptane permeance than that of traditional polyimide membranes (Starmem240) for OSN applications. Lively et al. further developed PIM membranes for effective organic solvent reverse osmosis.^[^ ^10^ ^]^ The PIMs have been highlighted as an ideal material to prepare high‐performance membranes for the separation in organic solvent systems. This is mainly because PIMs have high specific surface area, interconnected free volume elements, structural diversity, and flexible processability. The microporous nature of PIMs is achieved by introducing twisted molecular structures to inhibit the tight packing of chains. To maintain the formed micropores, a large number of rigid structures (e.g., aromatic rings and contorted structures) are required because they limit the rotational freedom of PIMs along the backbones, ensuring that the chains or segments do not rearrange their conformations. In addition, the rigid structures and strong intermolecular interactions play a crucial role in maintaining the overall free volume while mitigating excessive swelling in organic solvents, thereby ensuring a stable selectivity in organic solvents. However, there is unavoidable local tight packing of chains and/or segments in PIMs, resulting in numerous sub‐0.4 nm ultra‐micropores in the “interconnected pores”.^[^ ^11^ ^]^ Moreover, such sub‐0.4 nm ultra‐micropores are hard to control after synthesis due to rigid backbones. For glassy polymers with high fractional free volume (FFV), pre‐existing pores significantly influence solvent transport due to their relatively large average size and rigid structure, which is immobilized by an exceptionally high glass transition temperature (higher than decomposition temperature for PIMs).^[^ ^12^ ^]^ These sub‐0.4 nm ultra‐micropores are unfavorable for the efficient transport of solvent molecules due to their large kinetic diameters (i.e., usually ≥ 0.4 nm) and the tendency to transport in clusters,^[^ ^13^ ^]^ as shown in **Figure** **1**a. The existence of sub‐micropores is akin to a locally narrowed or blocked artery to impede blood flow. In this case, the permeation of solvents through PIM‐based membranes is far from ideal, especially compared with other microporous membranes, such as covalent organic framework (COF) membranes. Numerous strategies have been explored to obtain desirable solvent‐accessible pore structures or free volume to further advance the OSN performance of PIM membranes, including post‐polymerization modifications (e.g., side groups functionalization^[^ ^14^ ^]^ and crosslinking^[^ ^15^ ^]^) and post‐fabrication modifications (e.g., blending^[^ ^16^ ^]^ and solvent activation^[^ ^17^ ^]^). Although these methods are effective, they increase costs and impose limitations on the potential for scaling up in both production and applications. Finely engineering PIMs with intrinsic solvent‐accessible pore structures to fabricate membranes with high solvent permeability would offer broader opportunities to address the challenges. So far, PIM membranes with well‐tailored intrinsic free volume that offer rich accessible pores for solvent transport (achieved without post‐treatments), which can be more cost‐effective and easier to scale up, have not been reported for OSN applications.

Here, we report a microporous polyimide with high solvent‐accessible free volume for OSN to recover precious organic compounds in organic solvents. To tackle the crucial balance between enhancing intrinsic porosity for high permeability and maintaining the strong intermolecular interactions vital for solvent resistance, the design and synthesis of molecular stents in the microporous polyimide for the passage of organic solvents were elaborated, just like the images shown in Figure 1b. Further, its feasibility of using thin film composite membranes as OSN membranes was demonstrated. We sought to elucidate the role of the chemical structure on the free volume element of the polymer.

## 2 Results and Discussion

### 2.1 Polymer Design Based on the Nuanced Behavior of Aromatic Rings

The development of high‐performance OSN membranes hinges on achieving both high permeability, high selectivity and robust solvent resistance. In polymer membranes with high FFV and rigid pore structures, pre‐existing free volume elements play an important role in advancing the molecular transport of organic solvents for high permeability.^[^ ^12^ ^]^ However, the intricate impact of various chemical groups on the FFV within polymers is challenging to verify and quantify using conventional methodologies.

**Figure 2:**
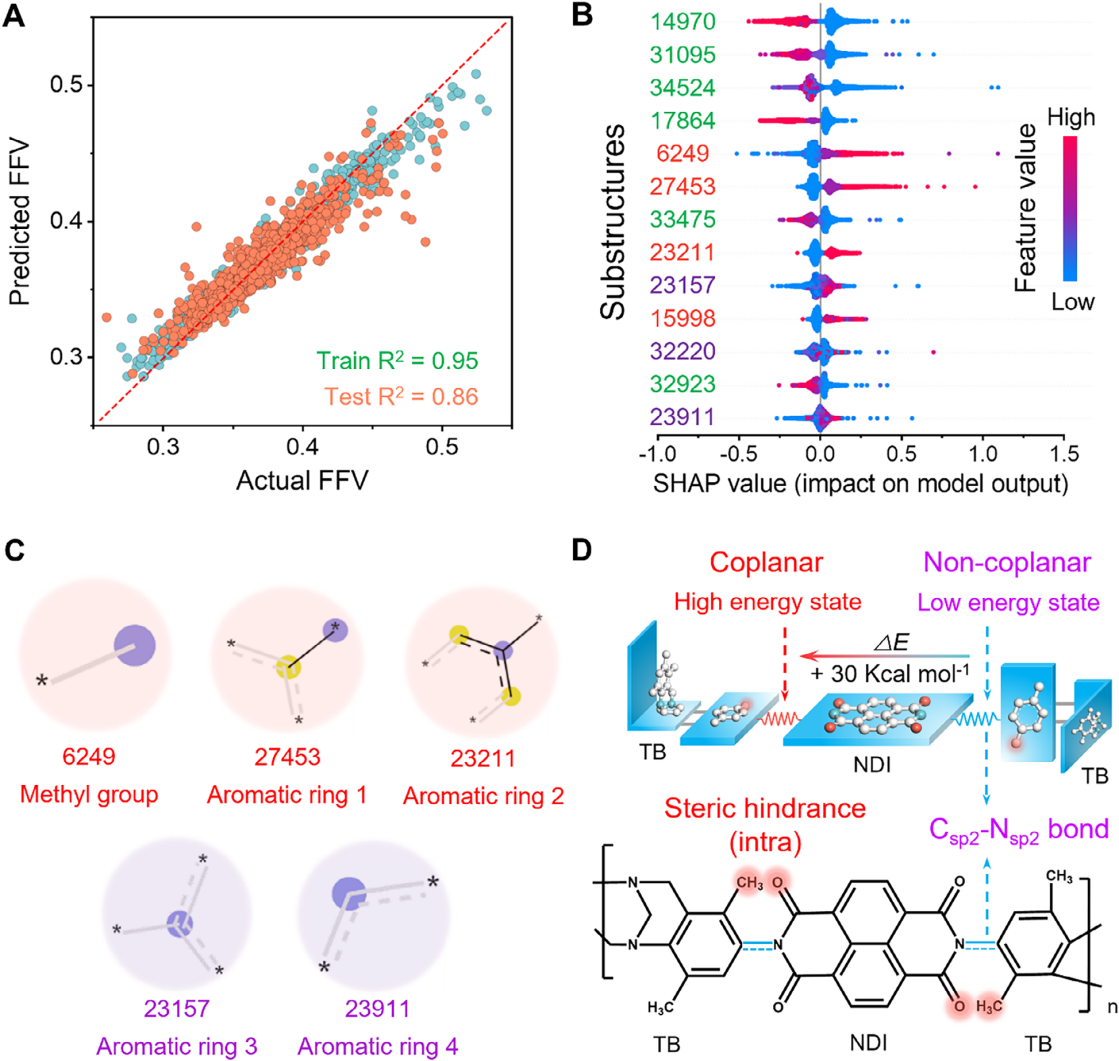
Impact of various chemical groups on FFV within polymers verified and quantified using machine learning. A) Predictive performance of the RF model. B) and C) Influence of top substructures on the FFV of polymers as predicted by the RF model. Each dot represented a substructure of a particular polymer in the dataset. The feature value indicated the impact degree of the substructure on FFV, and the color of the dots indicated the frequency of the substructure. Red text: positive effect on FFV; blue text: both positive and negative effects on FFV. D) Structural features of the PI‐TB‐NDI with ultra‐rigid non‐coplanar low‐energy state.

To accurately validate and quantify the role of the substructures on the FFV of microporous polymers, machine learning was employed (The code and datasets can be found at Section S1, Supporting Information), due to its ability to quantitatively predict complex and potential chemistry‐property relationships in materials, including recent advances in FFV prediction for polymer membranes.^[^ ^18^ ^]^ The 425 most frequent substructures were extracted from over 8000 polymers (see Table S1, Supporting Information for part of them) using the Morgan fingerprint with frequency (MFF),^[^ ^19^ ^]^ which included substructures (such as ─O─, ─CN, and ─CF_3_) commonly used in microporous polymers such as PIMs (Table S2, Supporting Information for details of how these substructures were captured, Table S3 (Supporting Information) for part of the substructures). We then established accurate polymer chemistry‐FFV relations utilizing the random forest (RF) model,^[^ ^20^ ^]^ as demonstrated in **Figure** **2**a ($R_{train}^{2}∼0.99,R_{test}^{2}∼0.87$) and Figure S1 (Supporting Information). Furthermore, the Shapley Additive exPlanations (SHAP) approach was used to interpret the RF model to assess the marginal contribution of each substructure on FFV.^[^ ^21^ ^]^ Figure 2b and Figure S2 (Supporting Information) present the influence of the top (i.e., the most important) substructures on the FFV based on the prediction of the RF model. Feature values and SHAP values were used to measure the number of substructures and their contribution to FFV, respectively. A substructure enhances FFV if higher feature values correlate with positive SHAP values, whereas it diminishes FFV when higher feature values align with negative SHAP values.

The analysis revealed that aromatic rings exhibit both positive and negative effects on FFV, in which the substructures of 27453 (aromatic ring 1) and 23211 (aromatic ring 2) showed highly positive effects and the substructures of 23157 (aromatic ring 3) and 23911 (aromatic ring 4) demonstrated both positive and negative effects (purple color in Figure 2b,c). Notably, the analysis highlights that although the positive influence is often dominant for many common aromatic groups, the often‐overlooked negative impact (likely due to enhanced packing efficiency) can be unexpectedly significant for certain configurations. We referred to this phenomenon as a “double‐edged sword” role of aromatic rings on free volume in determining dry‐state FFV. That is, the rigidity of aromatic rings could maintain high overall free volume, while stronger intermolecular interactions [e.g., *π*–*π* interactions and charge transfer complex (CTC)^[^ ^22^ ^]^] of aromatic rings promoted local tight packing, which reduces the intrinsic porosity of microporous polymers. In addition, we found that the methyl group (substructure 6249) is capable of increasing chain spacing and showed the second most important positive effect on FFV. This nuanced behavior of aromatic rings necessitates a balanced design strategy: maximizing the rigidity benefits they offer while mitigating their tendency to induce detrimental packing, aiming for higher FFV and intrinsic porosity.

However, translating these dry‐state FFV predictions to real‐world OSN performance requires further consideration. Although the strong intermolecular interactions potentially lead to denser packing (and lower predicted dry FFV), they become crucial to resist organic solvents. This is because these interactions bolster polymer chain cohesion, providing essential solvent resistance and structural integrity to prevent excessive swelling and maintain selectivity during operation. Consequently, a successful OSN polymer design must strategically leverage strong intermolecular interactions for bulk solvent resistance while concurrently creating and preserving accessible porosity for high permeability.

This presents a multi‐scale design challenge: how can we optimize structural features that enhance high intrinsic porosity without compromising the beneficial bulk interactions vital for solvent stability? Our strategy centers on inhibiting excessive local tight packing (which reduces permeability) while preserving the overall rigidity and cohesive forces imparted by the aromatic backbone. Informed by our ML analysis, we recognized that rigid polycyclic aromatic structures (like naphthalene, pyrene) enhance overall rigidity. The inhibition of excessive local packing while maintaining beneficial bulk interactions can be accomplished by substituting hydrogen atoms on aromatic rings with bulky groups (e.g., ‐CH_3_). Replacing some aromatic rings with alicyclic rings offers another potential route for regulating chain arrangements.

In this work, the extremely rigid naphthalenediimide (NDI) structure with fused aromatic rings was identified as the initial building unit due to its facile synthesis of microporous polymers. Its fused aromatic structure provides significant backbone stiffness and promotes strong intermolecular interactions, contributing to chain cohesion and solvent resistance. Here, Tröger's base (TB)^[^ ^23^ ^]^ was selected as the twisted building unit, given its high compatibility with NDI and its ability to alleviate undesirable overall tight packing through its high rigidity and contortion. Further, the ultra‐rigid NDI and TB units were linked by conjugation‐stiffened C_sp2_─N_sp2_ bonds^[^ ^24^ ^]^ to construct TB‐NDI‐TB building blocks to understand their role in suppressing the local tight packing, as illustrated in Figures 1b and 2d. If they can suppress local tight packing, we believe that these building blocks will act as molecular stents, minimizing local packing issues while preserving beneficial bulk interactions.

To facilitate the molecular‐level stents to inhibit local tight packing, an *ortho* (*o*) ‐methyl group (i.e., substructure 6249) on the imide ring was deliberately introduced onto the phenyl ring of TB. Note that the *o*‐methyl group enhanced the intra‐molecular steric hindrance between the TB and NDI by significantly increasing the torsional energy of the C_sp2_─N_sp2_ bond by 30 Kcal mol^−1^ (Figure 2d). Consequently, the aromatic ring of TB and the NDI planes could be perpendicular to each other, meaning that a more rigid and H‐shaped non‐planar conformation within the TB‐NDI‐TB units was formed. In addition, the *o*‐methyl group could enhance intermolecular steric effects to increase the chain spacing. Crucially, this “molecular stent” approach operates at a local scale, disrupting specific tight packing arrangements, while the bulk properties‐overall rigidity and intermolecular interactions from the NDI/TB backbone operate at the bulk polymer scale, ensuring overall solvent resistance. The two aspects are complementary to give OSN membranes high permeability and resistance, to address different requirements at different scales.

The predicted FFV of the PI‐TB‐NDI ranked as high as the top 20% among 21 microporous polymers with high FFV (Table S4, Supporting Information). Balancing features for bulk solvent resistance with localized structures intended to enhance accessible porosity, a PI‐TB‐NDI was determined as a microporous polymer favorable for separations in organic solvents. To demonstrate a negative control (Table S4, Supporting Information), when the NDI unit was replaced with a less rigid unit, such as an alicyclic unit, the predicted FFV significantly decreased to the bottom 50%. Furthermore, removing the methyl group from PI‐TB‐NDI to disable the molecular stents led to a sharp drop in the predicted FFV to the bottom 10%.

### 2.2 Synthesis and Properties of the PI‐TB‐NDI

PI‐TB‐NDI was synthesized using a polycondensation reaction of diamine monomer with dimethoxymethane (DMM) and was characterized in detail (Sections S2–S4, Supporting Information). Nuclear magnetic resonance (NMR) and Fourier‐transform infrared (FTIR) spectra confirmed the PI‐TB‐NDI synthesis. Three new signals appearing in the chemical shift range of 3 to 6 ppm were attributed to the methylene protons of the TB (Figure S3, Supporting Information). The C = O asymmetric stretching in the imide also appeared at 1716 cm^−1^, and the C─N stretching belonging to the imide was observed at 1331 cm^−1^ (Figure S4, Supporting Information). The number average molecular weight (M_n_) and polydispersity index (PDI) of the PI‐TB‐NDI were 157 kDa and 1.39, respectively (Figure S5, Supporting Information). Notice that the high molecular weight of the polymer contributed to its good film‐forming properties. Thermogravimetric analysis demonstrated that the PI‐TB‐NDI displayed convincing thermal stability with an initial decomposition temperature of up to 500 °C (Figure S6, Supporting Information).

**Figure 3:**
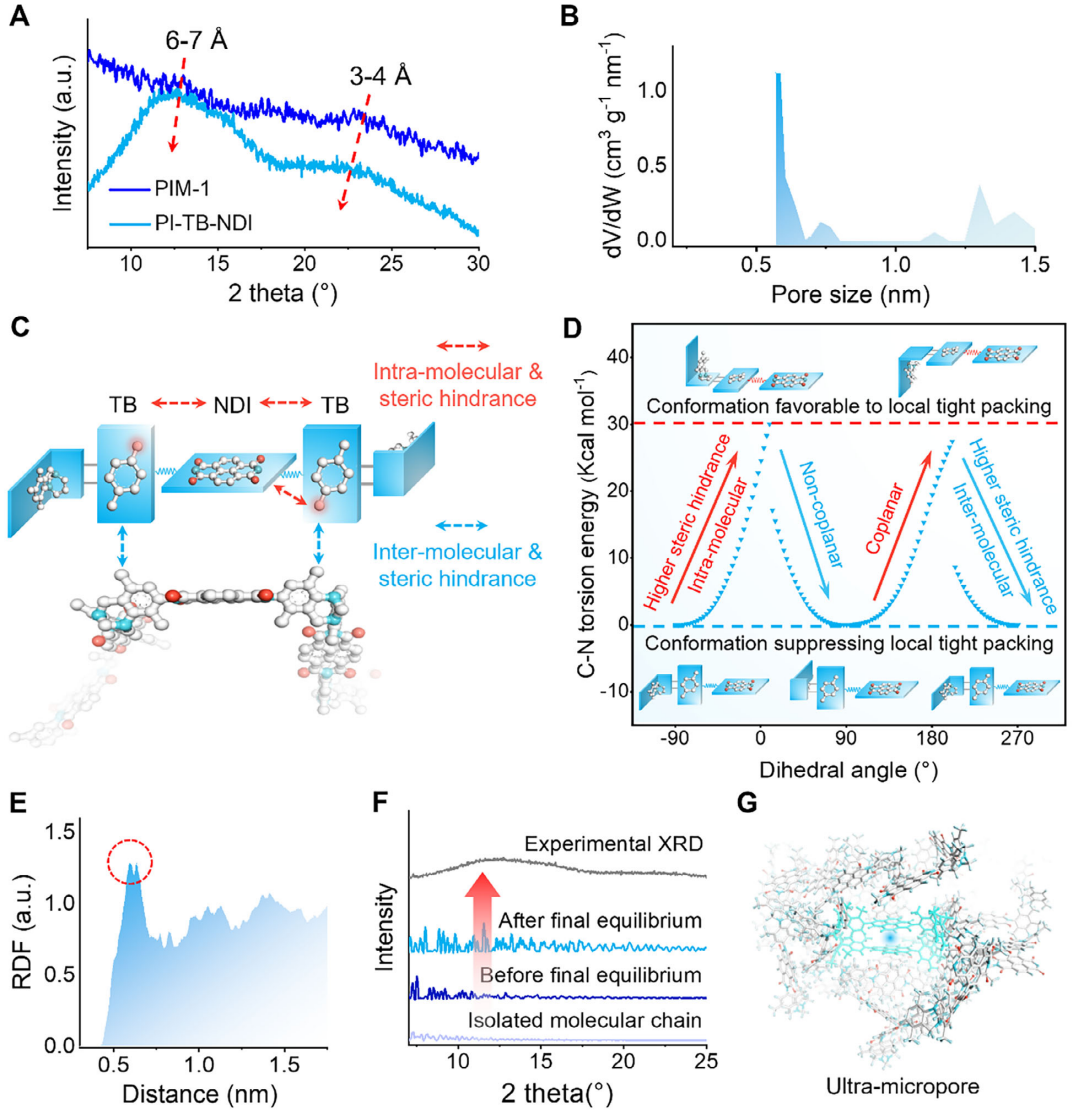
Chemical and physical structures of the PI‐TB‐NDI. A) Experimental XRD patterns. B) Experimental pore size distribution analyzed with the non‐local density functional theory method based on N_2_ sorption isotherms (slit‐pore geometry, carbon‐graphite model). c) Schematic diagram of molecular stent. D) Calculated C_sp2_─N_sp2_ bond torsion energy against the dihedral angle between the NDI and TB. The minimum energy point was assigned as 0 Kcal mol^−1^. The red arrows represent higher intra‐molecular and lower inter‐molecular steric effects, and the blue arrows represent the opposite. E) RDF curves of inter‐molecular chains for N atoms in NDI. RDF indicates the probability of finding an atom or a group at a certain distance from another atom or group. F) Simulated XRD patterns of isolated molecular chains and amorphous chain packing model containing 20 chains before and after final equilibrium (pack 1). The top one was the experimental XRD curve. The results of pack 2 and pack 3 are given in Figure S9 (Supporting Information). G) Typical ultra‐micropores (0.6–0.7 nm) in amorphous chain packing model.

The N_2_ adsorption isotherm demonstrated that the PI‐TB‐NDI had an attractive microporous feature with a Brunauer−Emmett−Teller (BET) surface area of up to 692 m^2^ g^−1^ (Figure S7, Supporting Information), remarkably higher than the PI‐ and TB‐based PIMs reported in literature (Figure S8, Supporting Information). Moreover, a sharp absorption at low pressure of p/p_0_ < 0.05 indicated a type I isotherm feature. From the X‐ray diffraction (XRD) pattern (**Figure** **3**a) of the PIM‐1 and PI‐TB‐NDI, the peak belonging to the d‐spacing of ultra‐micropores (0.6–0.7 nm) was displayed at 2θ ≈ 12.5°. In contrast, the d‐spacing of sub‐0.4 nm ultra‐micropores appeared at 2θ > 20°, which was smaller in the PI‐TB‐NDI compared with typical PIM‐1. Further, the pore size distribution (PSD) analysis using the non‐local density functional theory based on N_2_ sorption isotherms (Figure 3b) (which can detect ultra‐micropores of 0.6–0.7 nm and micropores) demonstrated that only the peak of the ultra‐micropores of 0.6–0.7 nm had an ultra‐high intensity and narrow distribution.

Note that the fraction of the sub‐0.4 nm ultra‐micropores in the PI‐TB‐NDI decreased because of the TB‐NDI‐TB limit local tight packing (Figure 3c). The key was the rotation of *o*‐methyl‐enhanced C_sp2_─N_sp2_ bonds to tailor intra‐ and inter‐molecular steric effects. The C_sp2_─N_sp2_ bond had a notable rotational energy barrier due to the *o*‐methyl groups in the TB unit (red dot in Figure 3c), which enhanced intra‐molecular steric effects to restrict its rotational flexibility, thus making TB‐NDI‐TB units with H‐shaped structures. The energy change during the rotation of the C_sp2_─N_sp2_ bond is detailed in Figure 3d. The horizontal axis denoted the dihedral angle between the TB and NDI connected by the C_sp2_─N_sp2_ bond. The vertical axis represented the conformational energy of the TB‐NDI structure. The conformational energy with the lowest value, assigned as 0 Kcal mol^−1^, served as the reference point for calculating other conformational energies. With the rotation of the C_sp2_─N_sp2_ bond, the intra‐ and inter‐molecular steric effects of TB‐NDI‐TB changed in exactly opposite ways, leading to a trade‐off effect between the intra‐ and inter‐molecular steric effects. The lowest intra‐molecular energy and steric effects were observed when the TB and NDI were perpendicular to each other (corresponding to the H‐shaped conformation of the TB‐NDI‐TB). Simultaneously, the inter‐molecular steric effects enhanced by *o*‐methyl groups were maximized and were favorable for limiting the inter‐molecular local tight packing. Conversely, when the TB and NDI were close to co‐planar conformation, the inter‐molecular steric effects were nearly minimized, while the intra‐molecular energy and steric effects were extremely high, suppressing the inter‐molecular local tight packing.

Therefore, the presence of both TB and NDI gave the PI‐TB‐NDI high overall free volume and high BET surface area with the increasing rigidity of rich aromatic rings. Furthermore, the local tight packing enhanced by *π*–*π* interaction and/or CTC was effectively suppressed within the PI‐TB‐NDI by introducing the *o*‐methyl groups in TB to form an H‐shaped conformation.

Molecular simulation (methodological details in Sections S5–S8, Supporting Information) was conducted to provide further evidence for the suppression of local tight packing. Figure 3e shows the radial distribution function (RDF) curve of inter‐molecular chains for N atoms in NDI units. A sharp peak appeared at ≈0.6 nm (i.e., the high probability of finding another NDI unit at a distance of ≈0.6 nm) in the RDF curve, indicating that the local tight packing between the NDI units was effectively limited. The simulated XRD patterns presented in Figure 3f depicts isolated molecular chains and an amorphous molecular chain packing model containing 20 chains before and after final equilibrium. The peak attributed to the ultra‐micropores (0.6–0.7 nm) was almost absent in the simulated curves of isolated molecular chains and amorphous chain packing model before equilibrium, but became prominent in the amorphous packing model after equilibrium. The intensity of the peak attributed to the ultra‐micropores (0.6–0.7 nm) increased significantly with high structure optimization levels and low system energy, ultimately matching well with the experimental XRD curves. These results suggested that the ultra‐micropores of 0.6–0.7 nm with high intensity and narrow distribution in Figure 3b were not derived from the intrinsically regular geometry of molecular chains. Instead, they were more likely to be advanced pore structures assembled in an orderly fashion by suppressing local tight packing and increasing chain spacing with TB‐NDI‐TB molecular stents in ultra‐micropores (0.6–0.7 nm), as shown by the detailed snapshot of molecular packing (Figure 3g). In this case, we expected the as‐developed PI‐TB‐NDI to achieve efficient transport of solvent molecules.

### 2.3 PI‐TB‐NDI Free‐Standing Ultrathin Film and its Thin Film Composite Membrane

**Figure 4:**
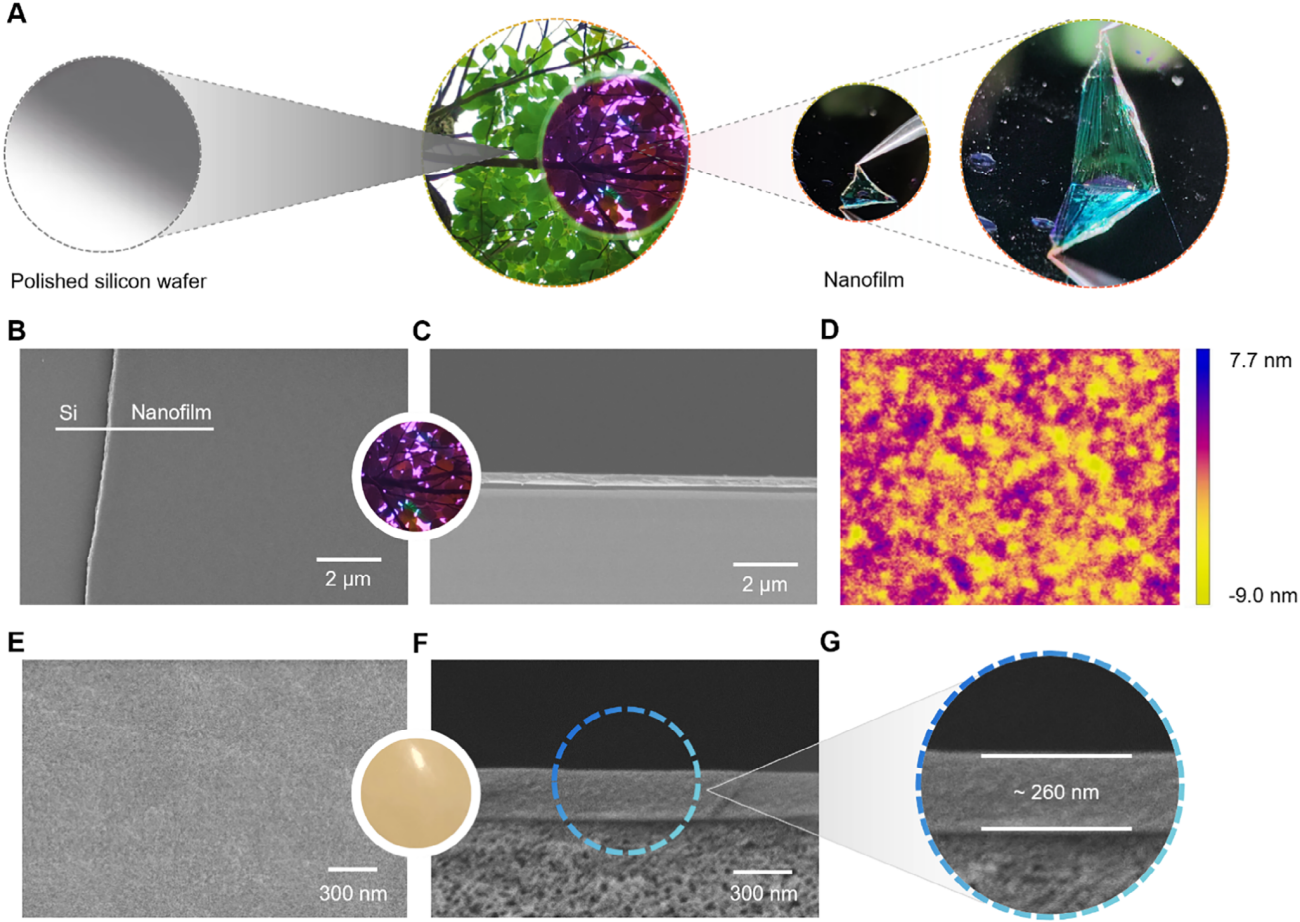
Morphologic images of PI‐TB‐NDI free‐standing ultrathin film and TFC membrane (Sections S11 and S12, Supporting Information). A) PI‐TB‐NDI free‐standing ultrathin film on a polished silicon wafer (1 wt.%, 2000 rpm). B) and C) Morphological images of the surface and cross‐section of free‐standing ultrathin film. d) AFM images of the surface of the free‐standing ultrathin film. e–g) Morphologic images of the surface and cross‐section of the PI‐TB‐NDI TFC membrane (0.75 wt.%, 2000 rpm).

The synthesized PI‐TB‐NDI exhibited excellent solvent processability in chloroform but showed strong solvent resistance to almost all commonly used organic solvents, such as dimethyl sulfoxide (DMSO), ethanol, methanol, heptane, acetone, ethyl acetate, and tetrahydrofuran (THF). As such, it was fabricated as a free‐standing ultrathin film using a spin coating method. **Figure** **4**a depicts a robust, transparent, and free‐standing PI‐TB‐NDI ultrathin film formed on a silicon wafer. Moreover, the ultrathin film could be peeled off from the silicon wafer using a tweezer without any obvious fracture, indicating a highly reliable strength for applications. The mechanical properties of this free‐standing film were further confirmed by a dynamic mechanical analyzer, and its tensile strength at break was ≈95.0 MPa, with an initial tensile modulus of 1.8 GPa, and elongation at break of 91.1%. A scanning electron microscope (SEM) image showed that the ultrathin film was completely defect‐free (Figure 4b) and tightly adhered to the silicon wafer with a thickness of ≈267 nm (Figure 4c). The atomic force microscope (AFM) images of the ultrathin film (Figure 4d; Figure S10, Supporting Information) showed that the intact ultrathin film was quite smooth and devoid of any defects, with an ultralow surface roughness of ≈2 nm. The ultrathin film thickness was also further confirmed to be ≈267 nm (Figure S10, Supporting Information) on AFM.

A PI‐TB‐NDI thin film composite (TFC) membrane was prepared using a crosslinked polyimide (XP84) ultrafiltration (UF) porous membrane as a substrate (Section S9, Supporting Information). The XP84 membrane had a typical asymmetric structure with an obvious porous surface with a pore size of ≈30–50 nm, showing a typical UF property (Figure S11, Supporting Information). However, to fabricate TFC membranes via a solution coating method, the polymer solution tends to penetrate the surface micropores of the substrate, which often leads to pore plugging and a significant reduction in membrane permeance. In addition, rapid evaporation of the solvent during the solution coating process results in a separation layer with surface defects. These issues limit the inherent performance of the separation layers, making it challenging to obtain robust TFC membranes that maintain the material's exceptional properties.

In this work, we successfully overcame these challenges and produced robust TFC membranes, leveraging the inherent performance of the PI‐TB‐NDI by constructing an oil‐water interface on the XP84 surface to limit the entry of water‐insoluble chloroform and PI‐TB‐NDI into the XP84 micropores. In addition, we used a new solvent vapor annealing (SVA) process to minimize surface defects (Section S10, Supporting Information).^[^ ^25^ ^]^ The morphologies of the resulting TFC membrane are presented in Figure 4e–g and Figures S12 and S13 (Supporting Information). By incorporating the SVA process, the ultrathin PI‐TB‐NDI film was quite free of defects on the XP84 membrane (Figure 4e; Figure S12a, Supporting Information), similar to that of its free‐standing one on a silicon wafer. Without using such a method, the membrane surface had large holes due to rapid solvent evaporation (Figure S12b, Supporting Information). On the other hand, the boundary between the PI‐TB‐NDI ultrathin film and the XP84 substrate was made very clear by engineering a water/oil interface (Figure 4g; Figure S13a, Supporting Information). The thickness of the separation layer based on the PI‐TB‐NDI ultrathin film was measured to be ≈260 nm, similar to the free‐standing ultrathin film despite variations in polymer concentration and surface tension between the fluid and substrate surfaces. The contact angles of ethanol and water were measured to be 25.1° and 77.7° (Figure S14, Supporting Information), respectively. In addition, the membrane was super‐wettable in other organic solvents (e.g., acetone and toluene), indicating that the PI‐TB‐NDI membrane was oleophilic and relatively hydrophobic in nature.

**Figure 5:**
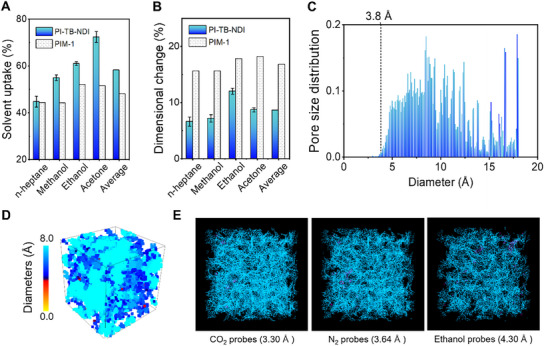
Solvent uptake and simulated structure in a solvent. A) Solvent uptake and B) dimensional change of PI‐TB‐NDI films in typical organic solvents at 25 °C and 1 atm. C) Calculated pore size distribution of equilibrated swollen PI‐TB‐NDI model (pack 1), with a simulated mass change of 61% determined by actual mass change. D) Visualized pore size distribution of equilibrated swollen PI‐TB‐NDI model, where the red, dark blue, and light blue areas represent sub‐0.4 nm ultra‐micropores (local tight packing), ultra‐micropores (0.6–0.7 nm), and micropores, respectively. The results of pack 2 and pack 3 are given in Figure S15 (Supporting Information). E) Calculated accessible (blue) and non‐accessible (magenta) surface area of equilibrated swollen PI‐TB‐NDI model (Pack 1) using three typical probe molecules. The results of pack 2 and pack 3 are given in Figure S16 (Supporting Information).

**Figure** **5**a shows the solvent uptake (or mass change) of the PI‐TB‐NDI film (≈150 µm, Section S11, Supporting Information) in organic solvents. Compared with PIM‐1 film, the PI‐TB‐NDI film demonstrated superior solvent uptake capability.^[^ ^8^ ^]^ This indicated that solvent molecules could penetrate into the porous PI‐TB‐NDI membranes and then fill it to a larger extent, a property that we speculate is a result of the formation of H‐shaped TB‐NDI‐TB molecular stents, which may enhance solvent accessibility and increase the overall free volume by suppressing local tight packing and promoting ultra‐rigid chain conformations. Solvent uptake can be positively related to solvent permeability.^[^ ^26^ ^]^ In addition, the PI‐TB‐NDI film displayed smaller dimensional changes (Figure 5b), indicating that further swelling of the polymer matrix was limited by the overall rigidity and intermolecular interactions from the NDI/TB backbone. This observation supports the hypothesis that the solvent uptake was mainly determined by pre‐existing interconnected free volume elements rather than additional free volume generated by polymer matrix swelling.^[^ ^27^ ^]^ The pre‐existing connected volume with a larger size and more connectivity could easily accommodate solvent molecules, thereby increasing solvent permeability without a significant change in chain packing and without the cost of reduced selectivity.^[^ ^28^ ^]^

To further illustrate the relationship between the pore structure and enhanced solvent accessibility, the swollen pore structure was simulated using a molecular simulation tool (Section S6, Supporting Information). The equilibrated swollen models exhibited pore size distributions (Figure 5c) with 99.69% of the pores larger than 4 Å (light and dark blue areas in Figure 5d), indicating that the local tight packing was effectively limited. As shown in Figure 5e, the accessible surface area (cyan area) of three typical probe molecules (CO_2_, N_2_, and ethanol) decreased only slightly as the probe diameter increased from 3.3 to 4.3 Å, while the inaccessible surface area (magenta) remained negligible.

### 2.4 OSN Performance of the PI‐TB‐NDI Membranes

The solvent permeation and solute rejection of the synthesized PI‐TB‐NDI TFC membranes were evaluated using a crossflow system by pressure‐driven filtration at 0.4 MPa for the initial screening of these membranes for OSN (Section S13, Supporting Information). The effect of preparation conditions (i.e., polymer concentration and spin speed) on the ethanol permeance (as a representative solvent) and the rejection of 1200‐Da‐Vitamin B12 (VB12, 1.4 nm in diameter) through the membrane is shown in Figure S17 (Supporting Information). Ethanol permeance through the membrane decreased with the increase in polymer concentration, while the VB12 rejection increased. The ethanol permeance increased with the spin‐coating speed, accompanied by a decrease in VB12 rejection. The TFC type membrane prepared by spin coating, dilute polymer concentration, and higher spin‐coating speed obviously reduced the separation layer thickness, thereby increasing the solvent flux. However, the resulting thinner membrane was susceptible to diminished strength, predisposing it to form through‐pore defects, ultimately compromising selectivity and operational stability. When the polymer concentration was 0.75 wt.% and spin‐coating speed was 2000 rpm, the membrane had a better overall performance, i.e., 7.7 L m^−2^ h^−1^ bar^−1^ of ethanol permeance and 98% of VB12 rejection. Such conditions were determined to prepare the PI‐TB‐NDI TFC membrane to further investigate the solvent permeance and solute rejection.

**Figure 6:**
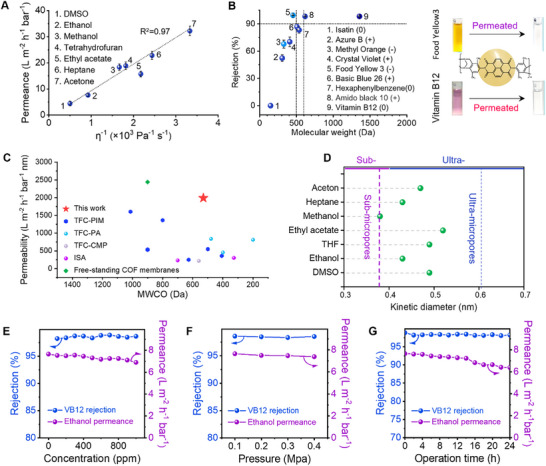
Solvent permeation and solute rejection performances of the PI‐TB‐NDI membrane. A) Effect of solvent viscosity on their permeation through the membrane. B) Solute rejection performance of the membrane and digital photos of the dye solutions before and after filtration by the membrane. Insets show the PI‐TB‐NDI chemical structure and its corresponding membrane digital photo. C) Performance comparison between the PI‐TB‐NDI membrane and the state‐of‐the‐art membranes reported in the literature. The Y‐axis is ethanol permeability. D) Kinetic diameter of solvent molecules and their permeation through sub‐0.4 nm ultra‐micropores and ultra‐micropores (0.6–0.7 nm). e–g) Effect of solute concentration, applied pressure, and operating time on membrane permeation and rejection performance.

The permeation of organic solvents of the PI‐TB‐NDI membrane (**Figure** **6**a) indicates that the order of permeance was DMSO < ethanol < THF < ethyl acetate < methanol < heptane < acetone (see Table S5, Supporting Information for detailed parameters of the organic solvent molecules). Notice that the product of permeance and viscosity was close to a constant, which was primarily governed by the pore‐flow model.^[^ ^29^ ^]^ This result confirmed that the as‐designed PI‐TB‐NDI membranes had relatively rigid pore structures, so that the FFV played a crucial role in governing the transport of organic solvents.^[^ ^12^ ^]^

The molecular weight cut‐off (MWCO) of the PI‐TB‐NDI membrane toward a total of four cationic and three neutral in ethanol was ≈500–600 Da, and that of two anionic dyes was 350 Da (Figure 6b; Table S6, Supporting Information). After filtration, the solutions changed from colorful to colorless. The high rejection of anionic dyes could be attributed to the combination of charge repulsion and size exclusion. Crucially, the inherent rigidity and strong intermolecular interactions derived from the polymer's aromatic backbone maintain a stable pore structure suitable for high selectivity, even under solvent‐induced swelling conditions. In addition, the densely distributed NDIs on the main chains have electron‐absorbing groups that generate *π*‐electron‐deficient structures (Figure S18, Supporting Information), which could form anion‐*π* interactions with a strong positive quadrupole moment and enhance rejection for negatively charged molecules.^[^ ^30^ ^]^

The performance comparison between the as‐designed PI‐TB‐NDI membrane and the state‐of‐the‐art membranes reported in the literature is summarized in Figure 6c and Table S7 (Supporting Information). Permeability (L m^−2^ h^−1^ bar^−1^ nm), which is independent of membrane thickness, describes the intrinsic permeation property of materials.^[^ ^9^ ^]^ The PI‐TB‐NDI membrane exhibited an extremely‐high permeability to commonly‐used solvents, i.e., 1990 L m^−2^ h^−1^ bar^−1^ nm for ethanol, 4770 L m^−2^ h^−1^ bar^−1^ nm for methanol, 5940 L m^−2^ h^−1^ bar^−1^ nm for heptane, and 8370 L m^−2^ h^−1^ bar^−1^ nm for acetone. Taking the example of ethanol, the permeability of 1990 L m^−2^ h^−1^ bar^−1^ nm significantly surpassed that of current microporous membranes, such as PIM‐1 (550‐1364 L m^−2^ h^−1^ bar^−1^ nm) and other PIM‐based (250‐1604 L m^−2^ h^−1^ bar^−1^ nm) TFC membranes (Table S7, Supporting Information). Although high permeability was achieved, a smaller MWCO (i.e., higher separation ability) could still be obtained. For comparison, the MWCO for free‐standing covalent organic framework (COF) membranes with similar ethanol permeability was almost two times higher (Figure 6c).^[^ ^31^ ^]^ This impressive achievement was largely attributed to high solvent‐accessible ultra‐micropores of 0.6–0.7 nm (Figure 6d) formed by the suppression of local tight packing due to the molecular stents of PI‐TB‐NDI. However, given that the size of formed ultra‐micropores (0.6–0.7 nm) was still smaller than that of most solutes (Table S5, Supporting Information) and further swelling (or penetrant‐induced glass transition^[^ ^27^ ^]^) was limited by the overall chain cohesion and the ultra‐rigid TB‐NDI backbone, the PI‐TB‐NDI membrane still remained very rigid and had high rejection toward solute molecules to show relatively small MWCO.

Figure 6e–g demonstrates the effects of solute concentration, applied pressure, and operation time on the solvent permeation and solute rejection performances of the PI‐TB‐NDI membrane. With an increase in VB12 concentration from 100 to 1000 ppm, the ethanol permeance and VB12 rejection of the membrane remained almost unchanged, i.e., only minimal pore blockage was observed (Figure 6e). This was mainly because the smooth membrane surface showed excellent anti‐fouling properties.^[^ ^32^ ^]^ When increasing the applied pressure from 0.1 to 0.4 MPa, the ethanol permeance was stable without obvious change (Figure 6f). Further, by prolonging the filtration time of VB12/ethanol solution to 24 h with crossflow measurements, the VB12 rejection was unvaried, while the solution permeance decreased slightly. This permeance decline was presumably a result of physical aging and compaction of the XP84 support.^[^ ^33^ ^]^ The findings provide a superb prediction to suitably bolster the potential of the membranes for widespread practical uses. Our next step is to make a module and investigate its ability to scale up in a large membrane area using crossflow mode permeation.

## 3 Conclusion

In summary, the solution‐processable microporous polyimide of PI‐TB‐NDI with intrinsically high solvent‐accessible free volume and high solvent resistance was synthesized, and the OSN membranes with high solvent permeability were prepared. These achievements were attributed to a well‐balanced molecular design: the specifically designed H‐shaped TB‐NDI‐TB molecular stents effectively inhibit detrimental local tight packing, thereby creating accessible porosity for high permeability. Simultaneously, the overall rigidity and strong intermolecular interactions derived from the bulk NDI/TB backbone are preserved, ensuring crucial solvent resistance and structural integrity. The conformation amplified sub‐0.4 nm ultra‐micropores to ultra‐micropores (0.6–0.7 nm), endowing the present microporous membrane with high solvent uptake, lower dimensional changes, and high intrinsic solvent‐accessible free volumes for rapid solvent transport. As such, the solvent permeability of the prepared PI‐TB‐NDI TFC membrane was significantly high. For instance, the ethanol permeability surpassed that of the traditional PIM membranes with similar selectivity by 2–8 times and was comparable to that of free‐standing COF membranes. Furthermore, due to the robust chain cohesion and ultra‐rigid backbone, it was highly resistant to almost all commonly used solvents and showed high permeability to them, such as 1990 L m^−2^ h^−1^ bar^−1^ nm to ethanol, 4770 L m^−2^ h^−1^ bar^−1^ nm to methanol, 5940 L m^−2^ h^−1^ bar^−1^ nm to heptane, and 8370 L m^−2^ h^−1^ bar^−1^ nm to acetone. Our work provides new guidelines for engineering microporous polymers with intrinsically well‐controlled pore structures without post‐treatments, enabling production scalability for upscaling and efficient solvent recovery, purification, and solute separation.

## 4 Experimental Section

Detailed experimental procedures, synthesis methods, characterization data, and original spectra are presented in the Supporting Information.

## Conflict of Interest

The authors declare no conflict of interest.

## Supporting information



Supporting Information

## Data Availability

The experimental and simulation data generated and analyzed in this study are available in the paper and/or its supplementary information. Machine learning dataset and code related to this study can be found on GitHub at https://github.com/AI4Polymer/FFV‐RF‐XGBOOST.
