# Supplementary material for: Microporous Polymer Membranes: Molecular Stents Enhanced Solvent‐Accessibility for Organic Solvent Transport
Source: Adv Sci (Weinh). 2025 May 29;12(30):e16748. doi: 10.1002/advs.202416748 (PMC12376686; doi:10.1002/advs.202416748)
Supplement: Supplementary file 1 — Supporting Information [file ADVS-12-e16748-s001.docx]

Supporting Information

Microporous Polymer Membranes: Molecular Stents Enhanced Solvent-Accessibility for Organic Solvent Transport

Shuang Guo, Chuanjie Fang*, Jiaqi Li, Xiaohe Wang, Weilin Feng, Hukang Guo, Ming Xie, Yongbing Zhuang, Young Moo Lee*, and Liping Zhu*

**Table of Contents**

Experimental Section/Methods

Results and Discussion

References

Author Contributions

**Experimental Section/Methods**

**Materials**

1,4,5,8-naphthalenetetracarboxylic dianhydride (NTCDA, 97.0%) and trifluoroacetic acid (TFA, 99%) were purchased from Tokyo Chemical Industry (TCI, Tokyo, Japan). Polyimide granules (P84) were purchased from HP Polymer GmbH (Austria). N-Methylpyrrolidone (NMP, 99.0%), dimethoxymethane (DMM, 99.0%), toluene (99.5%), ammonium hydroxide (25-28%), N, N-dimethylformamide (DMF, 99.5%), isopropanol (IPA, 99.7%), chloroform (CHCl_3_, 99.0%), dimethyl sulfoxide (DMSO, 99.0%), ethanol (EtOH, 99.7%), tetrahydrofuran (THF, 99.0%), methanol (MeOH, 99.5%), heptane (98.5%) and acetone (99.5%) were obtained from Sinopharm Chemical Reagent Co. (Shanghai, China). Isatin, Azure B, Methyl Orange, Crystal Violet, Crystal Violet, Food Yellow 3, Basic Blue 26, hexaphenylbenzene, and Amido black 10 were purchased from the Aladdin Industry Co. (Shanghai, China). Vitamin B12 (VB12) was purchased from Sigma-Aldrich Trading Co. Ltd. (Shanghai, China). The reagents were used as received without further purification.

**Method S1. Training of supervised machine learning models**

The dataset consists of 8045 homopolymers (**Table S1**) with their associated fractional free volumes (FFVs, calculated data from high-throughput MD simulations). These data were collected from the first large polymer FFV dataset developed by Li et al.^[1]^ The dataset they developed effectively addressing a gap in the PolyInfo database within this domain.

The repeating units of polymers were used to represent the polymers and labeled by their SMILES strings. Morgan fingerprints with frequencies (MFF)^[3]^ generated by RDKit^[4]^ captured the frequencies of the chemical parts (substructures) present in the repeating units (**Table S2** showed in detail how these substructures were captured), resulting in 425 different substructures (**Table S3**), which comprised the common structures found in PIMs.

The random forest (RF) model, which was faster and provides better interpretability (compared to neural networks), was trained to predict FFV based on the polymer chemistry, with 20% of the data reserved for the test set and the remaining 80% used for training. We trained multitask RFs using 100 estimators and a max tree depth of 30, with training capped at the square root (SQRT) of the number of features for each decision tree. Ten-fold cross-validation was used to tune the hyperparameters and select the best RF model (**Figure S1**). Then, this RF model was applied to predict the FFV of different PIM structures (**Table S4**).

Moreover, in-depth analyses of the data were conducted using the SHAP (SHapley Additive exPlanations) method on the best RF model, which provided interpretability to evaluate the impact of each feature or substructure on FFV (**Figure S2**).^[6]^

In addition, another machine learning model, the XGBoost model, was used to verify the credibility of the results. Both models demonstrated comparable performance and that 8 out of the 10 most important substructures were consistent across both models. This level of agreement between different models provided strong support for the importance of these specific substructures in predicting the FFV.

As a result, this combined approach of modelling and domain knowledge significantly enhanced our understanding of the underlying chemical phenomena, thereby confirming key molecular mechanisms influencing and regulating free volume in this work.

Machine learning datasets and code related to this study can be found on GitHub at https://github.com/AI4Polymer/FFV-RF-XGBOOST.

**Method S2. Synthesis of diamine monomer**

First, DPD (60 mol, 8.1720 g) and NTCDA (20 mol, 5.3636 g) were dissolved in 135 mL of N-methylpyrrolidone (NMP) and reacted at 90°C for 12 h to form the diamine monomer. Then, 50 mL of toluene was added into the three-neck flask, and the mixture was heated and refluxed at 90℃ for more than 9 h with a nitrogen atmosphere. Then, water was removed using a Dean-Stark trap. The reaction solution cooled to room temperature was poured into cold water to precipitate out the solid. The solid product was washed with water and then dried to obtain a red powdered diamine N. The crude product was recrystallized using ethanol and subsequently dried under vacuum at 80℃ for at least 12 h.


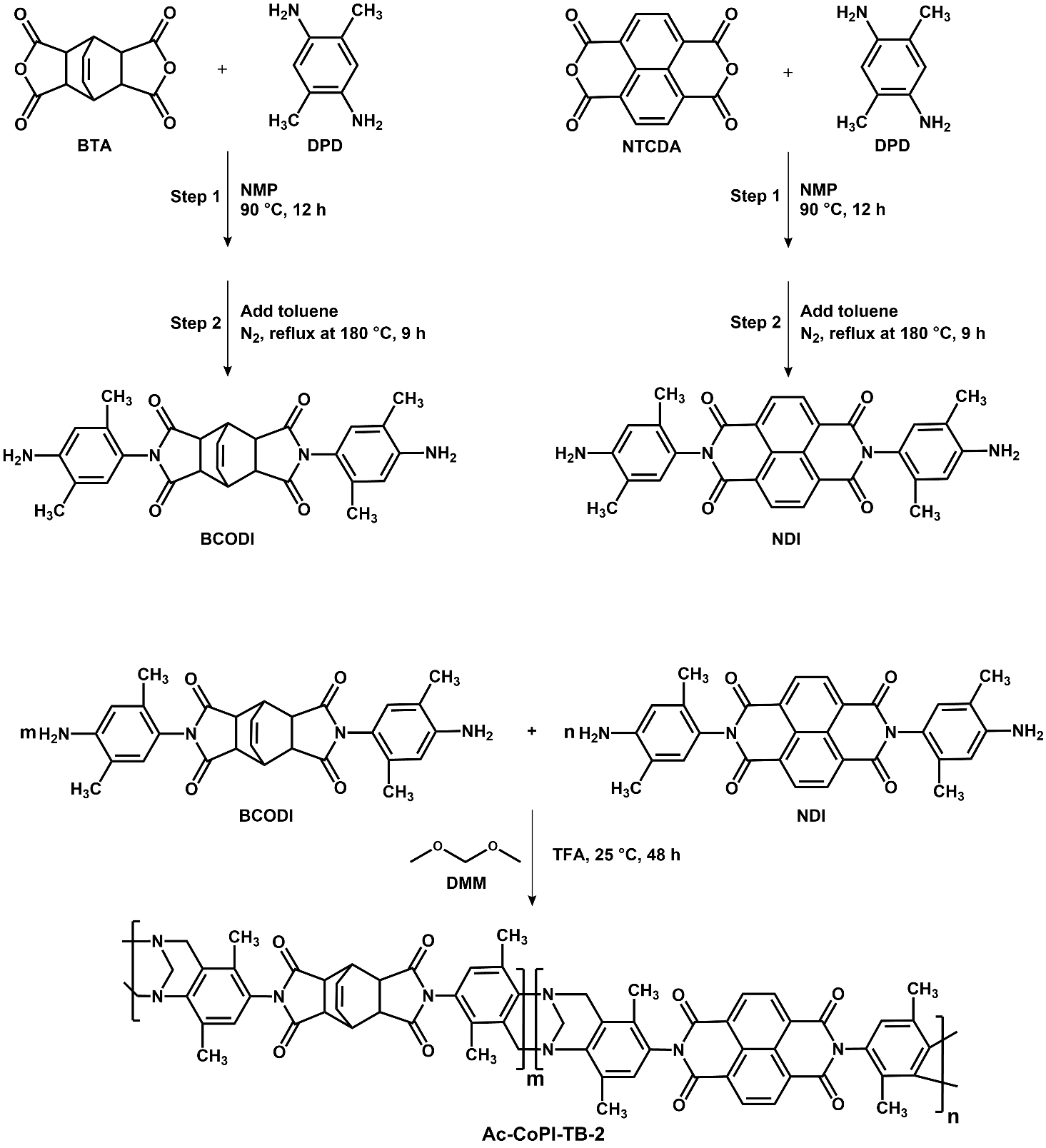


**Method S3. Synthesis of PI-TB-NDI**

The PI-TB-NDI was prepared by polymerizing DMM with the diamine N in trifluoroacetic acid (TFA). Diamine N (4 mmol) and DMM (22.4 mmol) were added to a three-necked flask, dissolved and then 30 ml of TFA was added drop-wise over 10 min. The reaction system was filled with nitrogen and stirred to obtain PI-TB-NDI at room temperature for 48 h, followed by alkalinization with a 2.5% aqueous ammonium hydroxide solution. The resulting yellow solid was washed three times with water and methanol, respectively. The product was purified by precipitation of a chloroform solution into methanol and dried in a vacuum oven at 120℃ for 24 h.


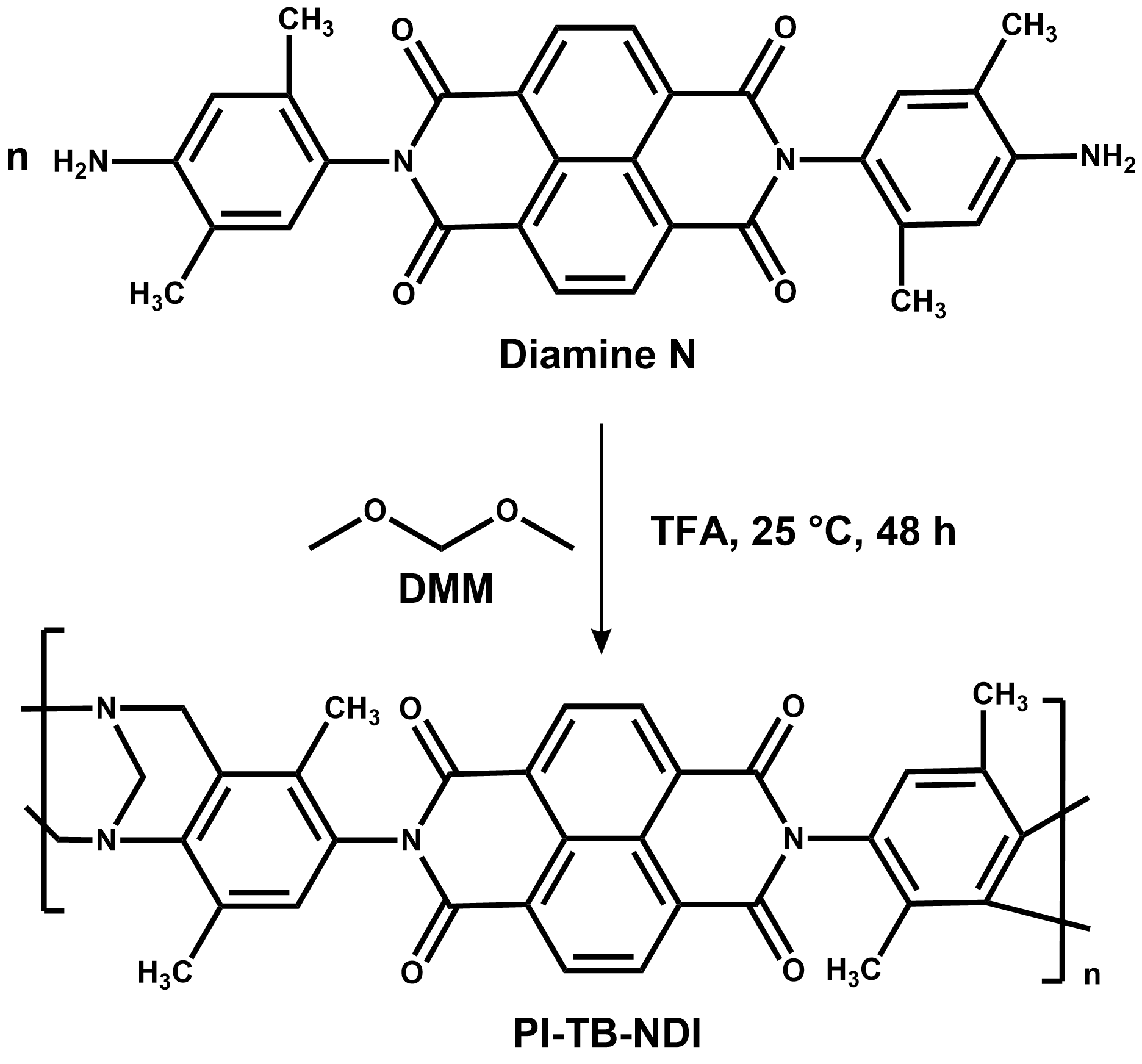


**Method S4. Characterization of the** **PI-TB-NDI with NDI and TB structures**

The molecular weight (M_n_ and M_w_) and polydispersity index (PDI) of the PI-TB-NDI were analyzed by gel permeation chromatography (GPC, Waters 1515, USA). The chemical structures of the PI-TB-NDI were analyzed by solution-state ^1^H nuclear magnetic resonance spectroscopy (NMR, Bruker Advance III 500, Germany) and attenuated total reflectance Fourier transform infrared spectroscopy (ATR-FTIR, Nicolet 6700, USA). The nitrogen sorption isotherms of the PI-TB-NDI at 77 K were analyzed by an automatic specific surface and porosity analyzer (ASAP 2460, USA). The chain spacing of the PI-TB-NDI and PIM-1 was analyzed by X-ray diffraction (XRD, X-pert Powder, Netherlands), and the interchain d-spacing was calculated using Bragg’s law. The thermal stability and glass transition temperature of the PI-TB-NDI were analyzed by a simultaneous thermal analyzer (STA, TGA/DSC3+, Swiss) with a heating rate of 10 K min^−1^ under a nitrogen atmosphere of 50 mL min^−1^.

**Method S5. Polymer model generation**

The structure with three repeat units was optimized by Gaussian 09 at the B3LYP/6-311G* level of theory ^[7]^. Restrained electro static potential (RESP) charge was calculated for the optimized structure using Gaussian 09 and Multiwfn version 3.8(dev).^[8]^ Three independent amorphous models of the PI-TB-NDI were built with Material Studio (MS) version 2019^[9]^ provided by BIOVIA. The amorphous cell tool was used to set 3D periodic boundary conditions while growing the molecular chains inside the three-dimensional box segment by segment, with random torsion, using a Monte Carlo moves algorithm. The probability of the Monte Carlo algorithm was calculated according to Flory's Rotational Isomeric State (RIS) theory.^[10]^ The initial packing procedure of amorphous chain packing models was performed with a template chain of 10 monomers and each packing model contained 20 polymer chains (13440 atoms) at an initial box size of 7.23×7.23×7.23 (nm) and an initial density of 0.4 g cm^3^. All initial packing models obtained in this way were subsequently equilibrated by a 21-step molecular dynamics equilibration using the GROMACS version 2023.1^[11]^ with the GAFF^[12]^ force field, which was generated by Sobtop version 1.0(dev 3.1).^[13]^ The final box size was 5.75×5.75×5.75 (± 0.01) nm and the final density was 0.98 ± 0.01 g cm^−3^.

**Method S6. Computational Polymer Swelling**

Each annealed model of PI-TB-NDI was artificially swollen with a mass change determined by the actual mass change (61%) of PI-TB-NDI dense film soaked overnight in ethanol. Specifically, 1472 ethanol molecules were inserted into the above-mentioned initial packing model with a box size of 7.23×7.23×7.23 (nm) and an initial density of 0.4 g cm^3^. All initial packing models containing ethanol molecules obtained in this way were subsequently equilibrated by a 21-step molecular dynamics equilibration using the GROMACS. The final box size was 6.56×6.56×6.56 (± 0.01) nm.

**Method S7. Polymer model analysis**

The simulated XRD curves were evaluated using Materials Studio's “Reflex” modules, and the radial distribution function (RDF) for N_sp2_ atoms in NDI units were analyzed in Materials Studio's “Forcite” modules. The RDF is typically utilized to quantify the frequency of occurrence of an atom within a given region.^[14]^ The porosity analysis (pore size distribution, accessible surface area) was performed by zeo++ version 0.3, and this method was chosen due to the presence of permanent microporosity within rigid PIMs.^[15,16]^ The pore size distribution was calculated using a probe diameter of 2.4 Å. The accessible and non-accessible surface area were visualized for 3.3 Å, 3.64 Å and 4.3 Å probe diameters.

**Method S8. Density functional theory (DFT) calculations**

A potential energy surface scan involving C_sp2_-N_sp2_ bond rotation was performed on Gaussian 09W with B3LYP/6–311G*. The empirical correction for dispersion interaction (DFT-D3(BJ)) proposed by Grimme was included to deal with van der Waals and some other dispersion attractive interaction forces.^[17]^ GaussView 5.0 software was employed to visualize the data obtained.

**Method S9. Preparation of XP84 ultrafiltration membranes**

A casting solution (22 wt% P84) was obtained by dissolving P84 with DMF. Then, the solution was poured on the smooth side of polyester non-woven backing attached to the glass plate by a casting knife with a gap of 250 μm. The polyester non-woven backing attached to the glass plate was horizontally immersed in 30°C deionized water (coagulation bath) for 5 min to form P84 membrane by phase inversion. Finally, the p84 membrane was cut into 5×5 cm pieces and put into IPA for 6 h to remove water. After that, the 5×5 cm P84 membranes were crosslinked with 1,6-hexanediamine (20 g L^−1^ in IPA) at 25 ℃ for 16 h under air bath oscillation. Finally, the XP84 membrane was immersed in deionized water for solvent exchange to remove IPA and excess 1,6-hexanediamine, and the XP84 membrane was stored in deionized water for later use.

**
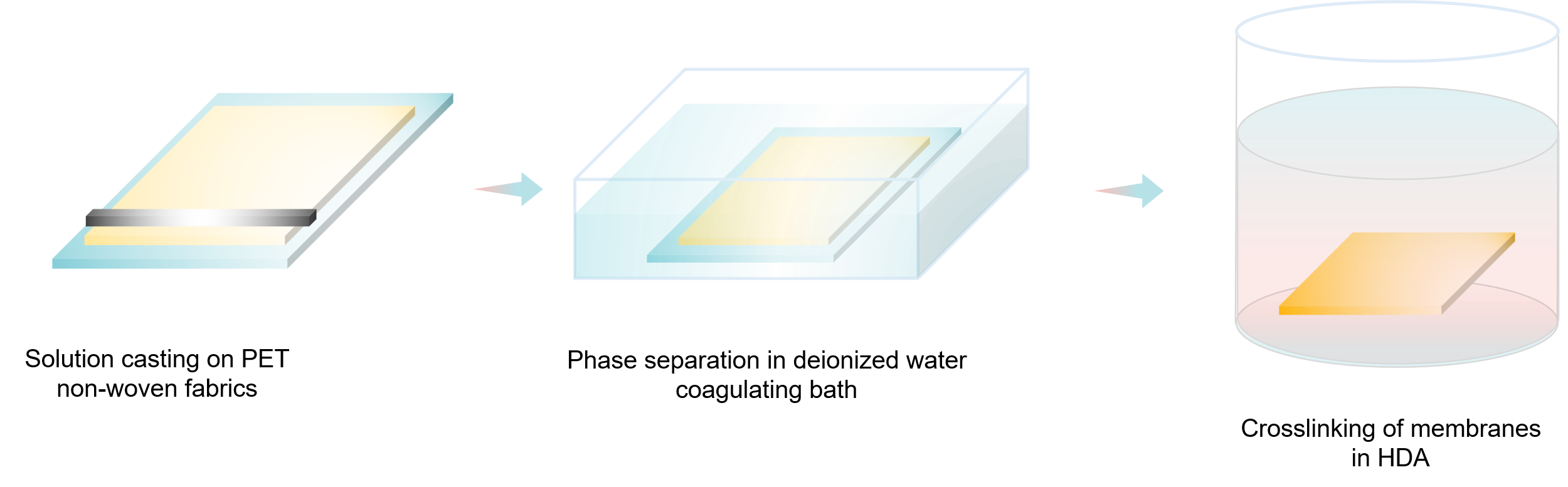
**

**Method S10. Fabrication of TFC membranes and free-standing thin films**

The PI-TB-NDI TFC membranes were formed using an improved spin-coating method followed by a SVA. The XP84 support membrane was first immersed in deionized water for 24 h to form an oil-water interface with chloroform on the surface afterwards, thus blocking the molecular chains from penetrating into the micropores of XP84 to avoid plugging. Then, it was placed onto a specially made microporous substrate and fixed by a vacuum pump. Chloroform was added to the center of the support membrane drop by drop, and the spin-coater was started according to the preset parameters (500 rpm for 3 s and 2000 rpm for 40 s, 25℃) to fill the spin-coating cavity with chloroform vapor and form an oil-water interface on the surface of support membrane. Similarly, PI-TB-NDI dissolved in chloroform with a pre-determined concentration (0.5-1.5 wt%) was poured onto the support membrane. A thin layer was formed on the top of the support membrane after imposing rotation with preset parameters (500 rpm for 3 s and 2000 rpm for 40 s, 25℃). The semi-dry membrane was kept in a spin-coating cavity filled with chloroform vapor to conduct a SVA process (2 min, 25℃) to eliminate remaining interfacial defects, which had a profound impact on the morphology and performance of final TFC membranes. Finally, the PI-TB-NDI TFC membrane was taken out and dried in air (60 min, 25℃) before being soaked into ethanol for storage. Similarly, the free-standing thin films were prepared using polished silicon substrates (1 wt% , 500 rpm for 3 s and 2000 rpm for 40 s, 25℃).


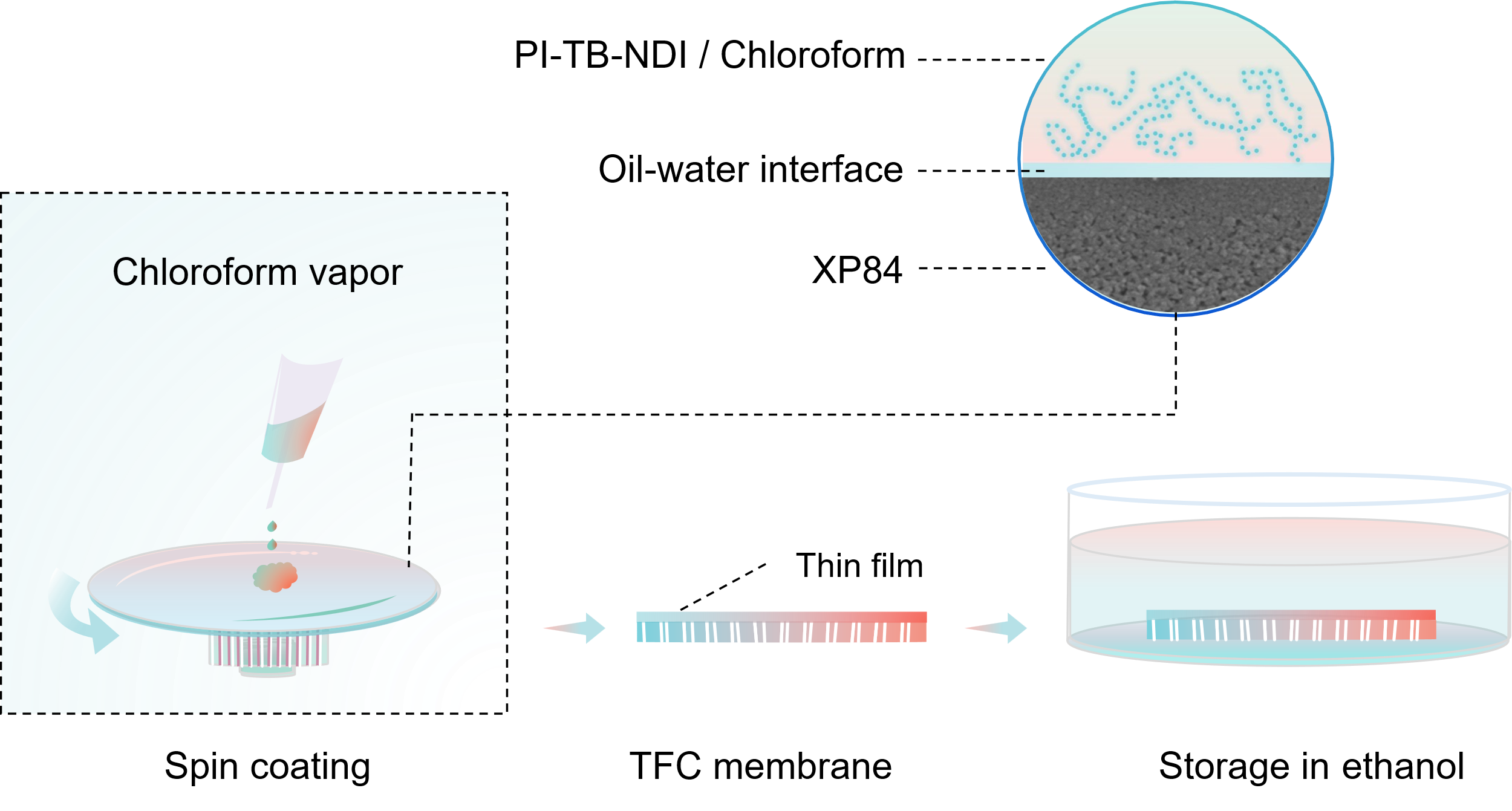


**Method S11. Fabrication of TB-NDI-TB films and swelling experiments**

PI-TB-NDI dissolved in chloroform with a concentration of 2 wt% was cast in a circular flat-bottomed glass dish and evaporated slowly at ambient temperature and atmospheric pressure for 3 days. Then, the dry dense films were soaked in methanol overnight and dried at 120℃ under vacuum for 12 hours. A dynamic mechanical analyzer Q800 (TA Instruments, USA) was used to measure the mechanical properties of the films.

The solvent resistance of PI-TB-NDI in DMSO, ethanol, THF, methanol, heptane, acetone, and ethyl acetate was determined by immersing the completely dried dense film of known mass in the selected solvent overnight and recording the change in mass after redrying at 120℃ under vacuum for at least 12 hours. Similarly, the swelling degree of PI-TB-NDI was determined by recording the mass and length increase of the completely dried dense films after overnight immersion in the selected organic solvents.

**Method S12. Characterizations of the** **free-standing ultrathin films and TFC membranes**

Their surface and cross-section morphologies were observed by field-emission scanning electron microscopy (FE-SEM, SU−8010, Japan). The surface morphological fluctuation of the films was observed by atomic force microscopy (AFM, MultiMode, USA). A contact angle measuring system (OCA-20, DataPhysics Instruments, Germany) was used to test the water and ethanol contact angle of the membrane.

**Method S13. Membrane performance measurements**

The permeation and rejection performance of the PI-TB-NDI TFC membranes was evaluated by a crossflow system driven by a plunger pump (LC-3060B, China). The membrane was pre-pressured with pure solvent under 4 bar for 1 h to reach a stable permeance. Subsequently, the pressure was kept constant to measure the membrane permeance. The membrane permeation performance, *J* (L m^–2^ h^–1^ bar^–1^), was evaluated using seven organic solvents (**Table S5**) as calculated with Equation (1):

$J=\frac{V}{A\cdot\Delta t\cdot P}$ (1)

where *V* (L) is the solvent permeation volume during the operating time *Δt* (h), *A* (m^2^) is the effective membrane area, and *P* (bar) is the transmembrane pressure.

To express the ability of the material to allow molecules to pass through, permeance was normalized with thickness to obtain permeability (L m^–2^ h^–1^ bar^–1^ nm). Permeability was defined as permeance multiplied by thickness and was a thickness-independent property ^[18]^. According to the Hagen-Poiseuille model ^[19]^, i.e., Equation (2), for the same solvent and constant transmembrane pressure, the product of permeance and thickness is only related to the structure of the material, i.e., porosity, pore size, and tortuosity:

$J_{v}=\frac{\varepsilon r_{p}^{2}}{8l\tau}\frac{\Delta p}{\eta}$ (2)

Here, *J_v_* is the solvent velocity through the membrane pores, and *η* is solvent viscosity. Membrane material is represented by porosity, ε, pore size, *r*_p_, tortuosity, τ, and membrane thickness, *l*.

The membrane rejection performance was evaluated using a series of probe molecules (anionic dyes, cationic dyes, and neutral molecules, **Table S6**). Ethanol was used as a solvent, and the concentration was 20 ppm. The membrane was pre-pressured with solution under 4 bar for 24 h to reach a stable rejection performance. The solute concentrations in the feed and permeate solutions were detected using a UV/vis spectrophotometer (UV5500-PC, shanghai Jingke Instrument, China). The rejection (R,%) was calculated using Equation (3):

$R=\left( 1-\frac{C_{p}}{C_{f}} \right)\times100\%$ (3)

Here, C_f_ and C_p_ represent the dye concentrations in the feed and permeate solutions, respectively. Each of the reported solvent permeance and rejection was an average value obtained by three parallel measurements, and the standard deviation was also reported.

Results and discussion


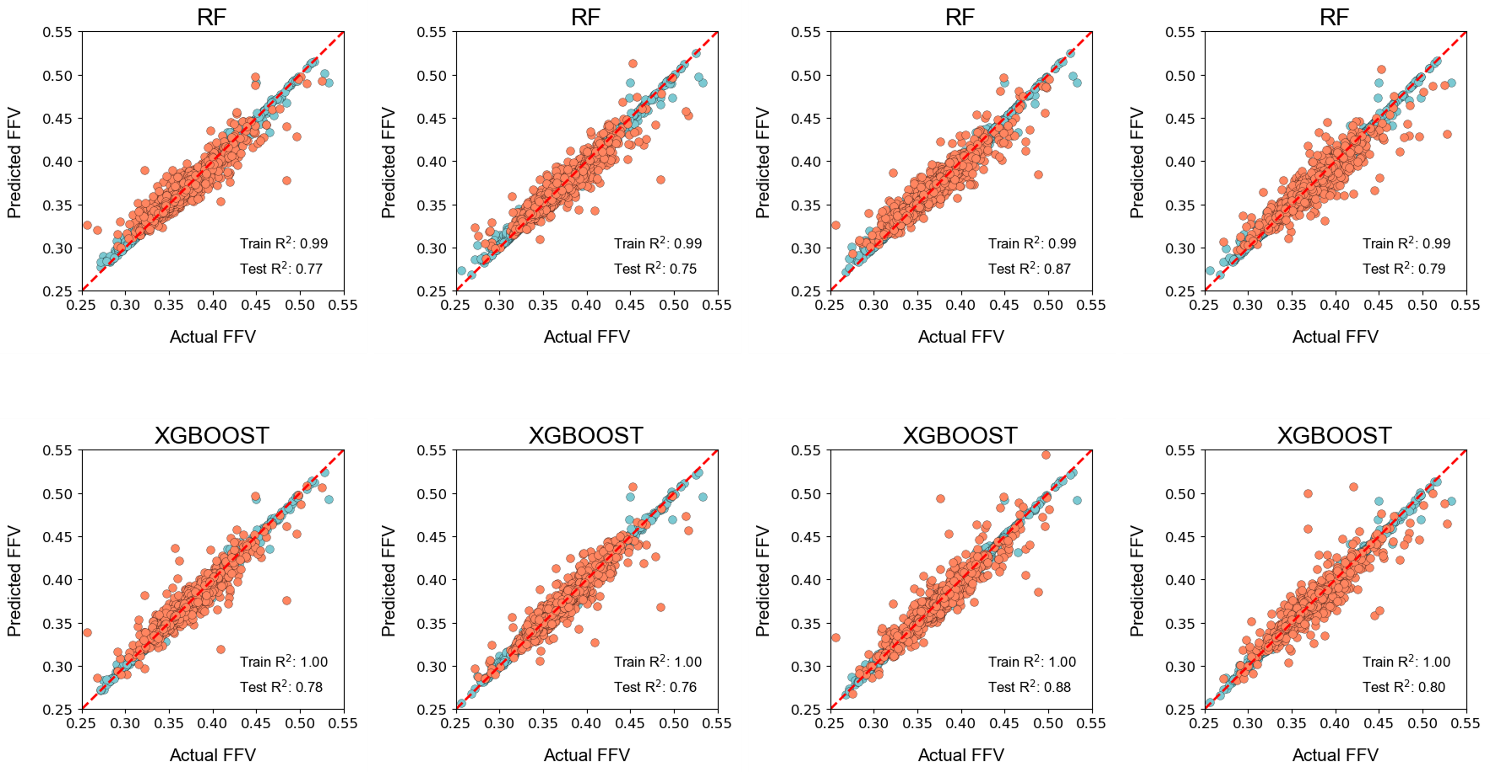


**Figure S1.** Performance of the RF and XGBoost models. The four subplots resulted from using different training/test sets with random seeds equal to 6, 16, 26, 36 respectively.


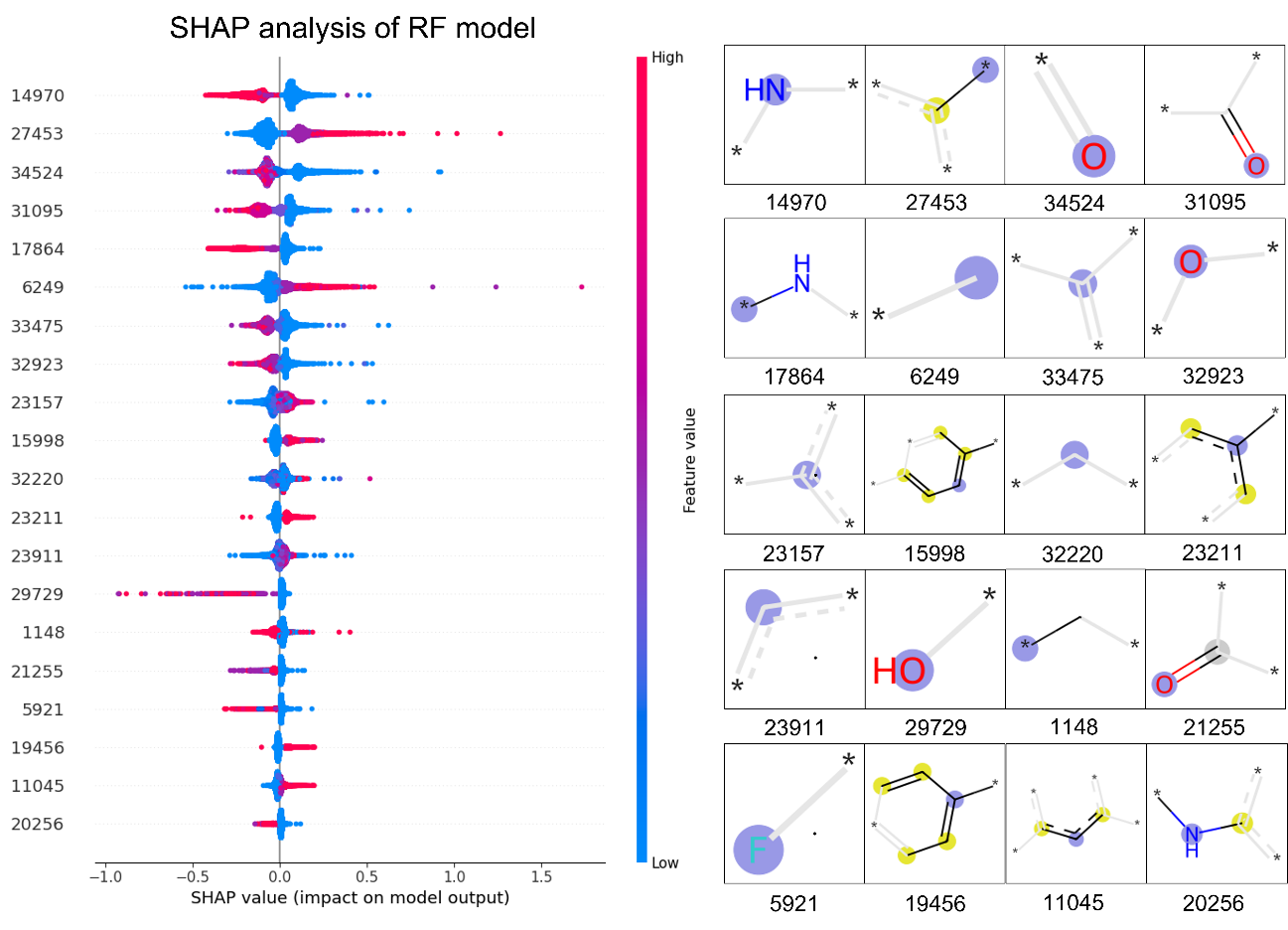


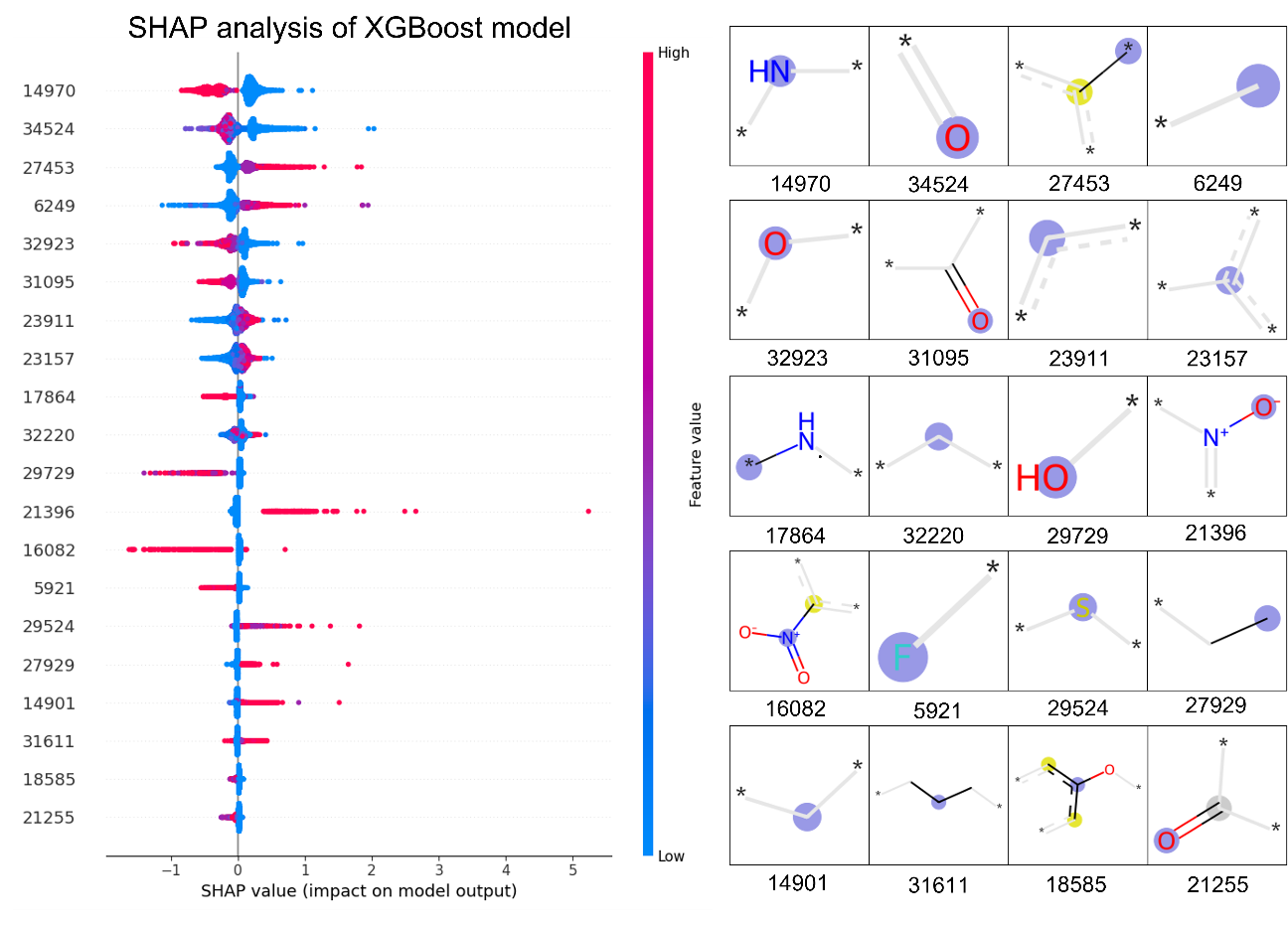


**Figure S2.** Results of SHAP analysis based on RF and XGBoost models and the twenty most important substructures.


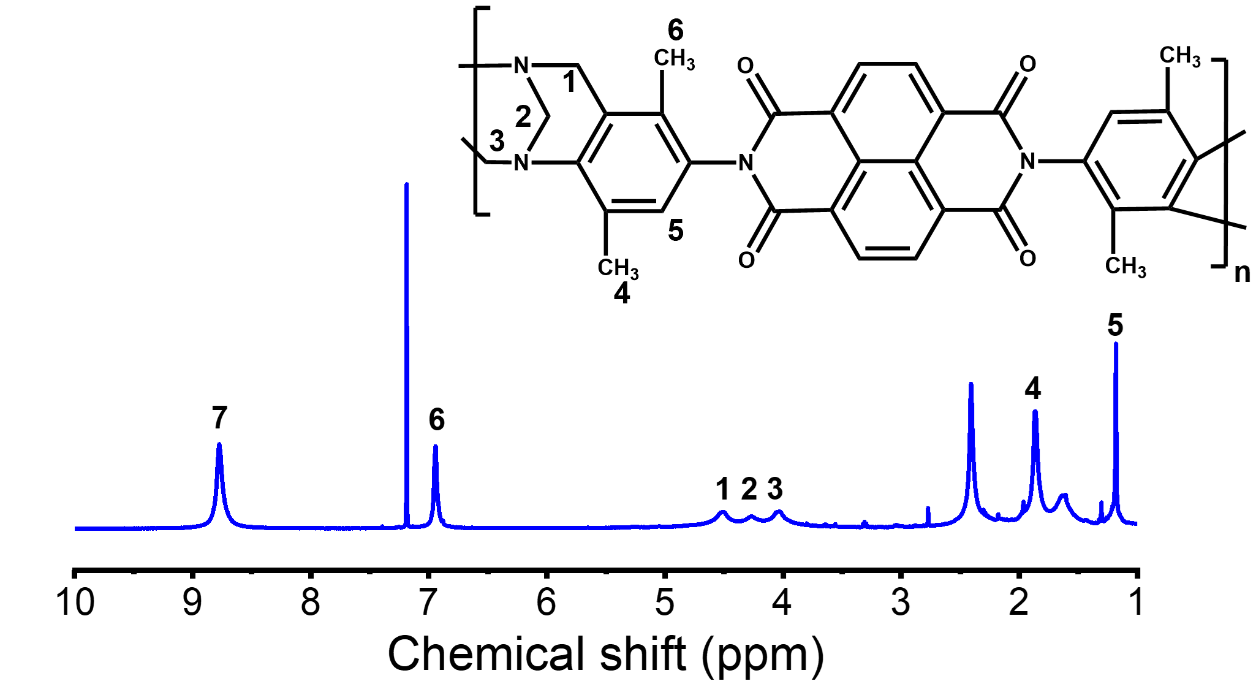


**Figure S3.** ^1^H NMR spectra of PI-TB-NDI. Three new signals (1, 2 and 3) appearing in the chemical shift range of 3 to 6 ppm confirmed the successful formation of the Tröger’s base structure.

**
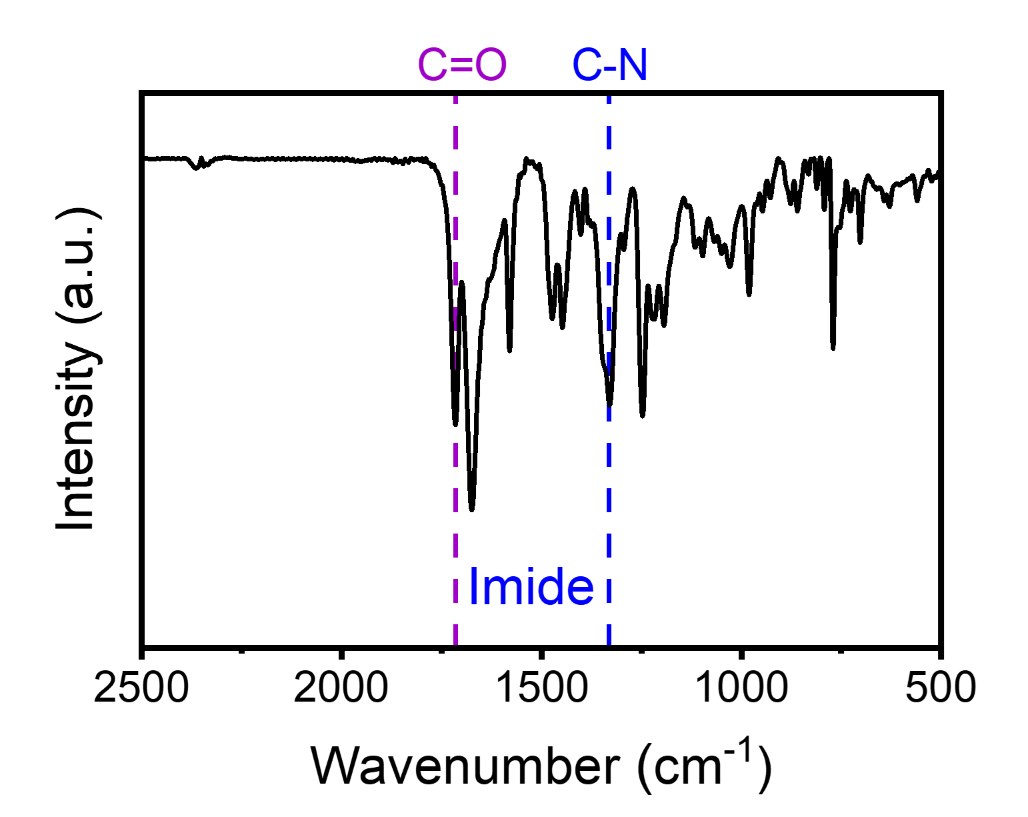
**

**Figure S4.** FT-IR spectra of the PI-TB-NDI (1719 cm^–1^, imide carbonyl asymmetric stretching; 1331 cm^–1^ imide −C−N).


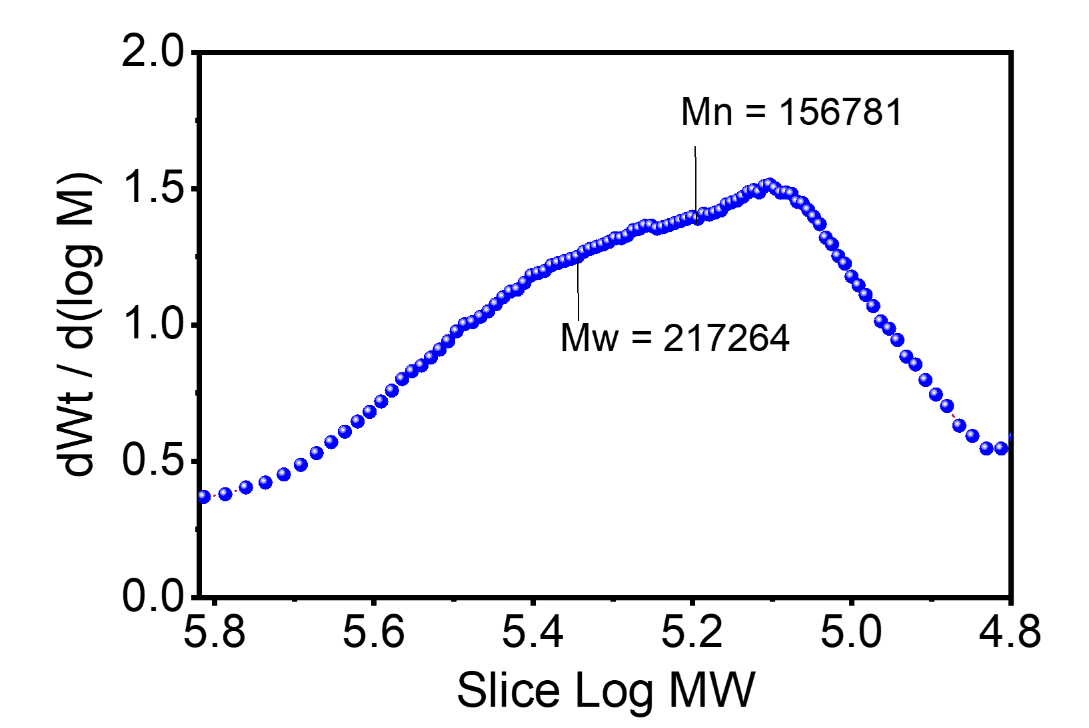


**Figure S5.** GPC spectrum of the PI-TB-NDI polymer.


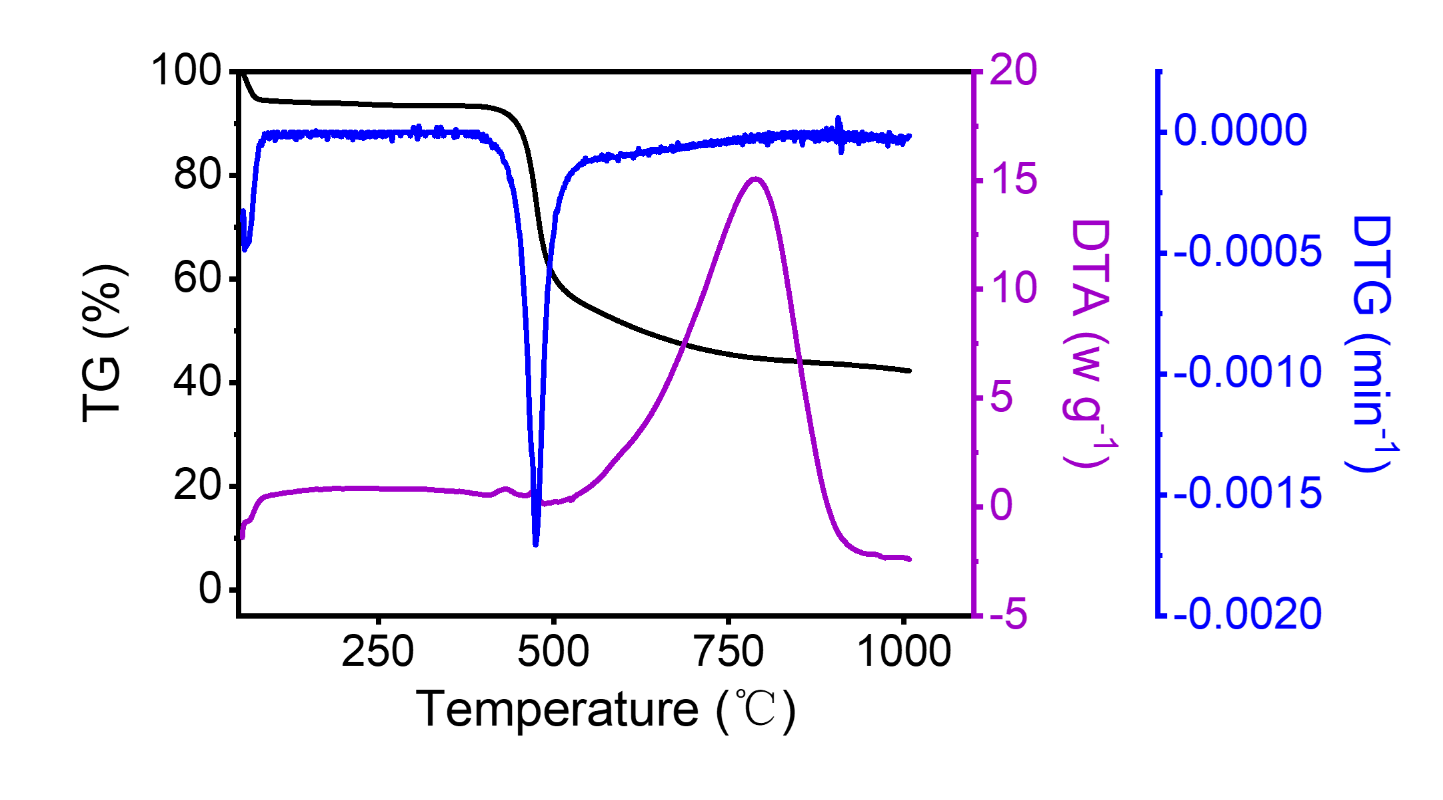


**Figure S6.** Thermal analysis of the PI-TB-NDI polymer.


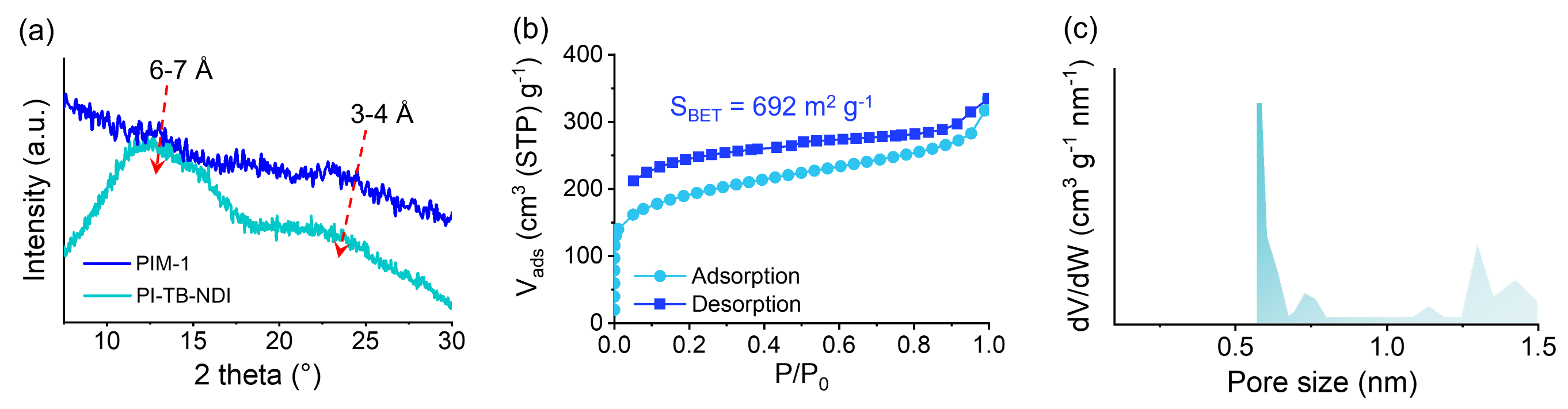


**Figure S7.** N_2_ adsorption−desorption isotherms of the PI-TB-NDI polymer at 77K.


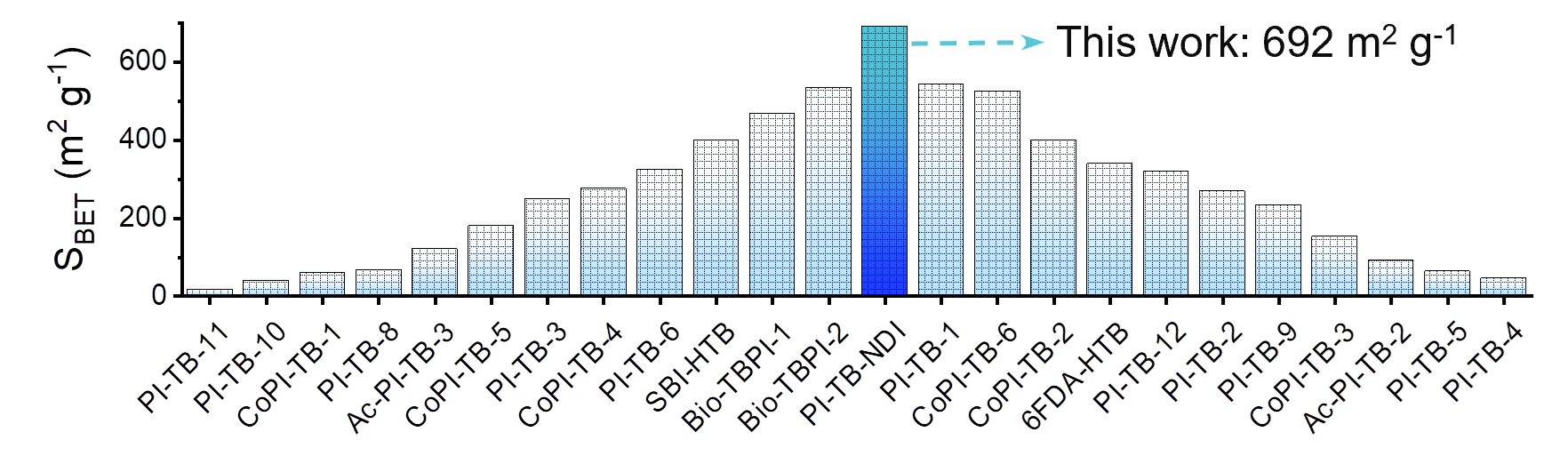


**Figure S8.** S_BET_ of the PI-TB-NDI compared with other PI- and TB-based PIMs.^[20–26]^

**
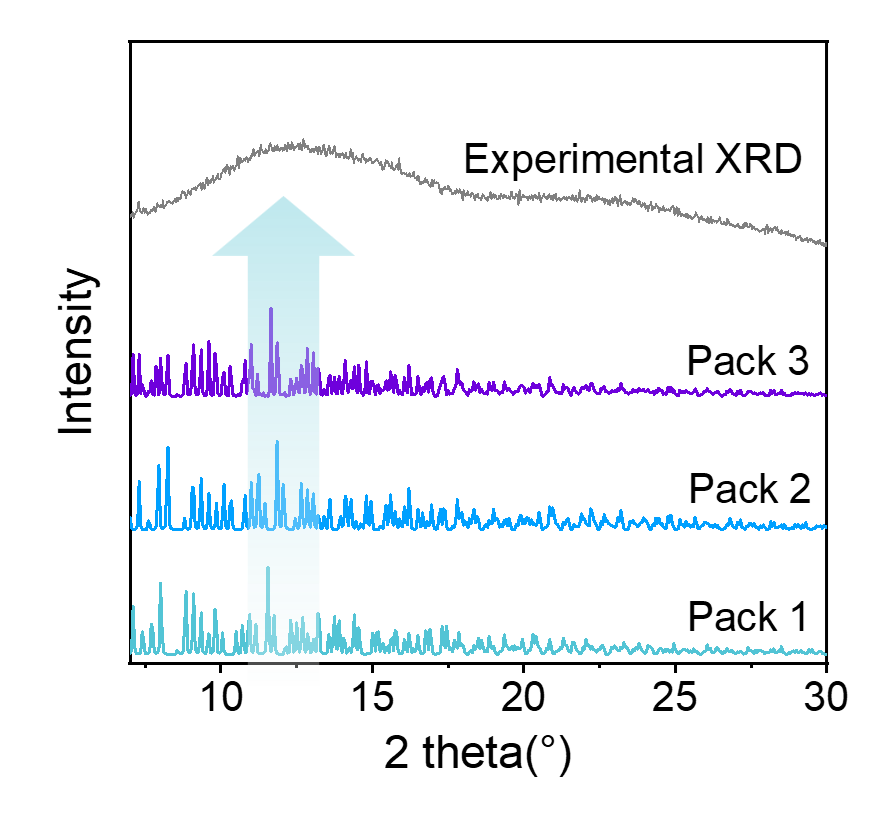
**

**Figure S9.** Simulated XRD patterns of amorphous chain packing model containing 20 chains after final equilibrium. Packs 1-3 represent three independent models to adequately sample different structures.


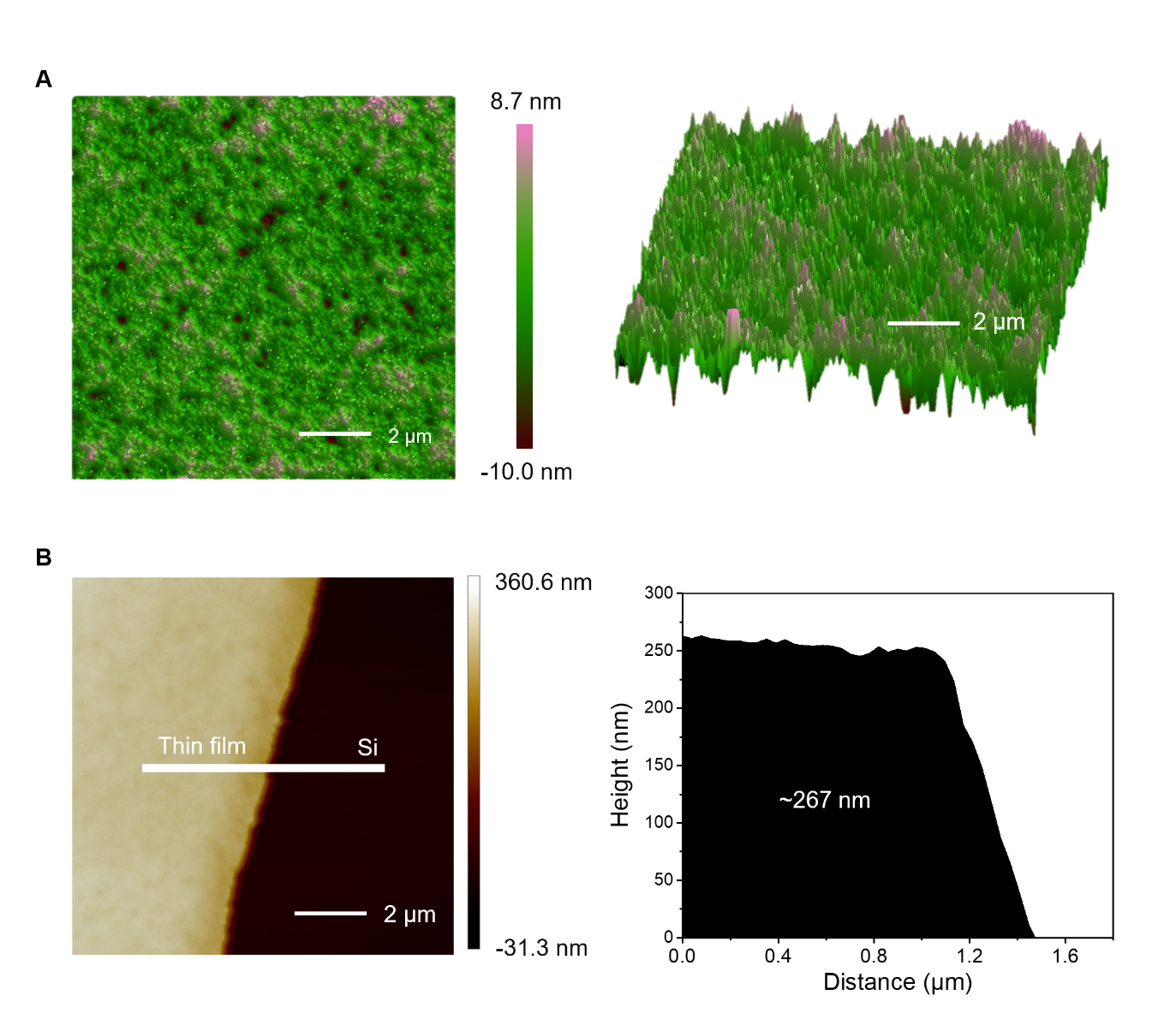


**Figure S10.** AFM images of PI-TB-NDI thin film. a) AFM surface roughness of the nanofilm (R_a_ = 2.04 nm). b) AFM height image of the nanofilm.

**
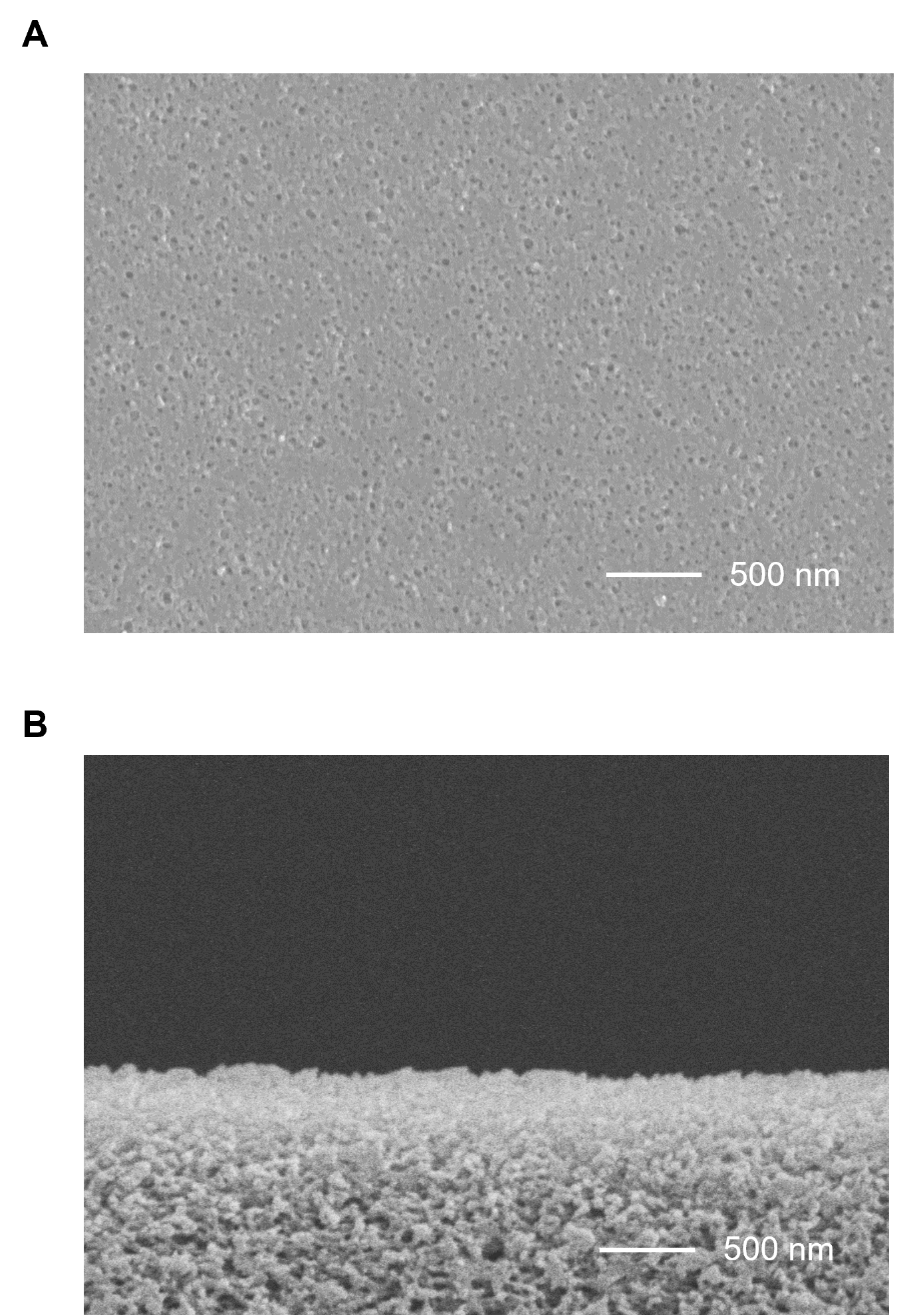
**

**Figure S11.** SEM images of the XP84. a) surface and b) cross-section images.


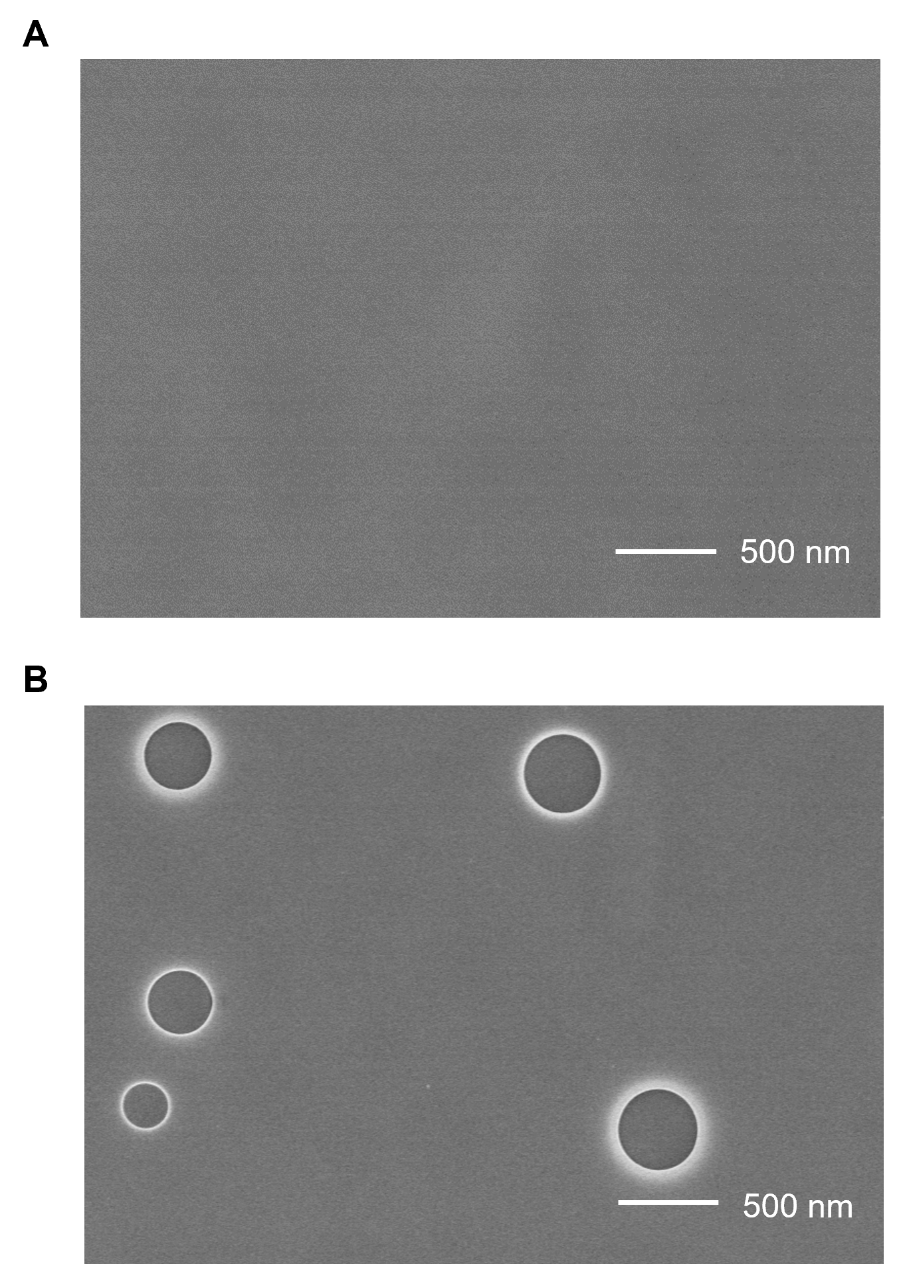


**Figure S12.** Surface SEM images of the TFC membrane. a) With SVA process and b) without SVA process (circled areas are surface defects due to rapid evaporation).

`
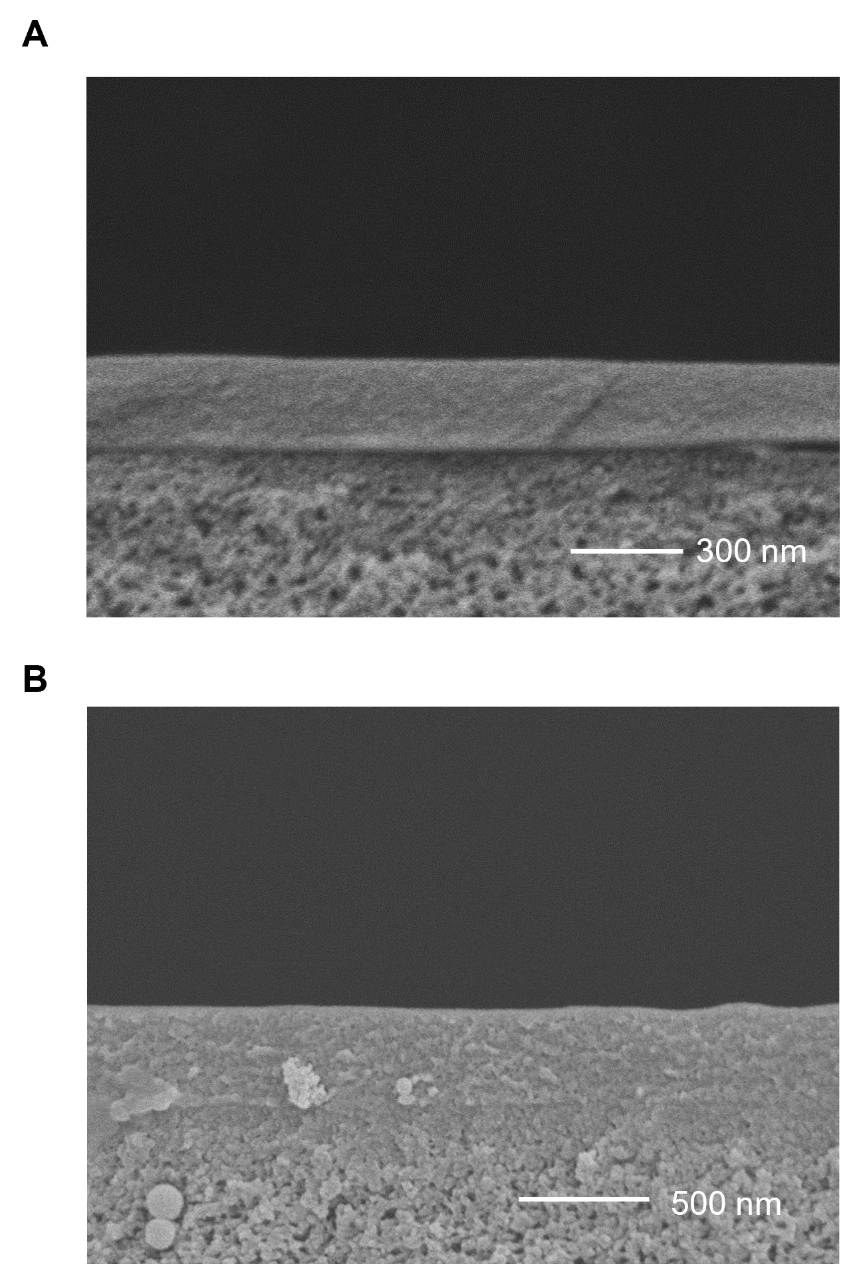


**Figure S13.** Cross-sectional SEM images of the TFC membrane. XP84 support membrane treated with a) deionized water and b) ethanol.

**
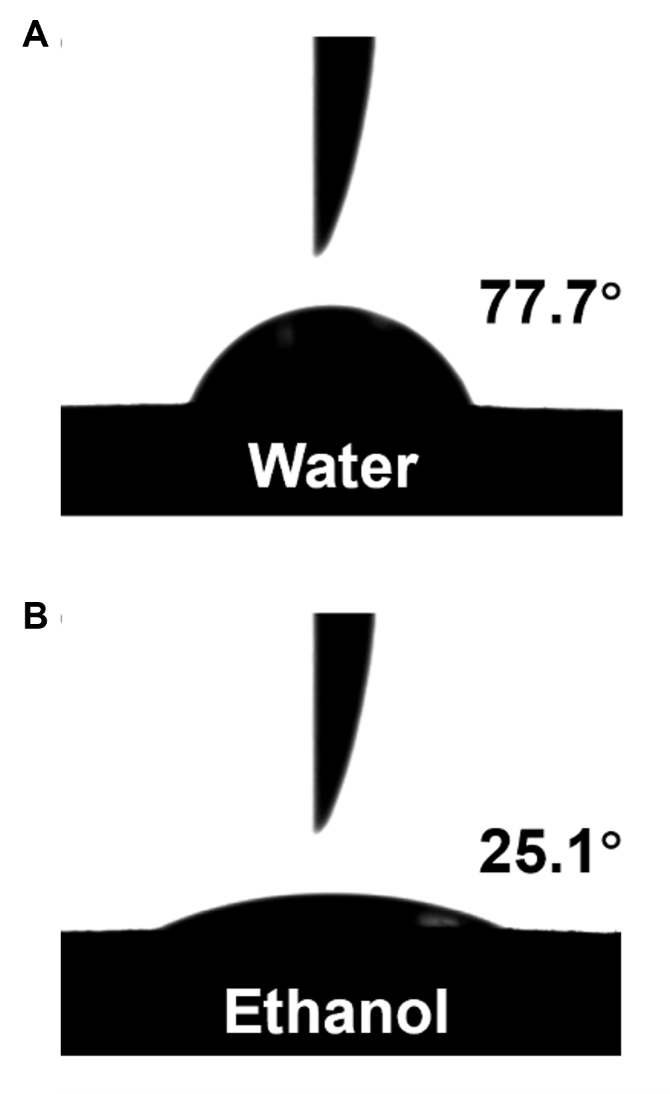
**

**Figure S14.** Contact angle of the PI-TB-NDI film. a) water contact angle and b) ethanol contact angle.


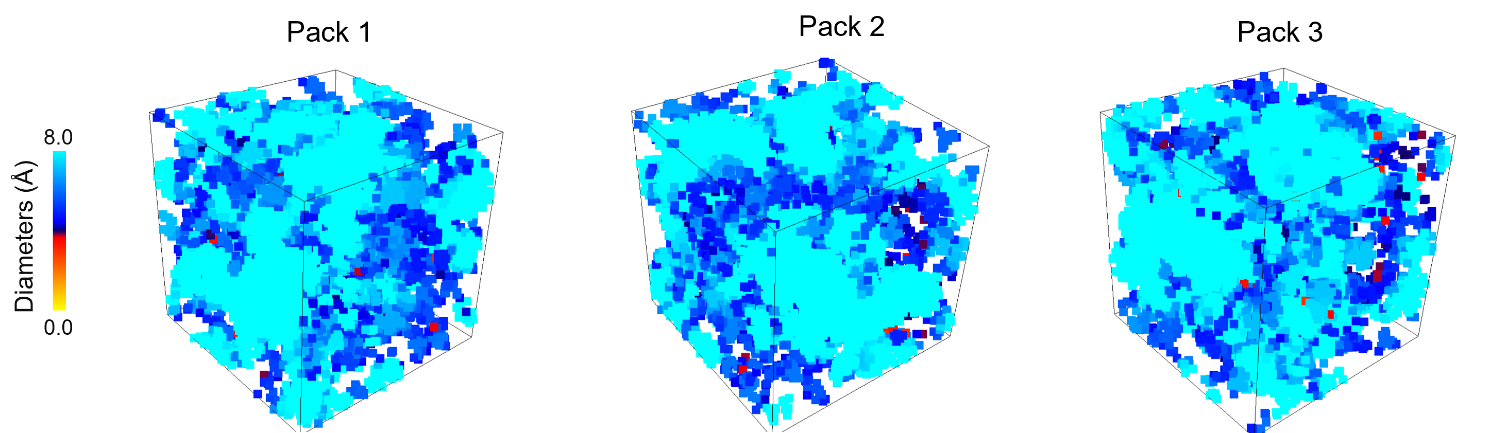


**Figure S15.** Visualized pore size distribution of equilibrated swollen PI-TB-NDI models (packs 1-3), where the red, dark blue, light blue areas represent sub-micropores (local tight packing), ultra-micropores (0.6-0.7 nm), and micropores, respectively. Packs 1-3 represent three independent models to adequately sample different structures.


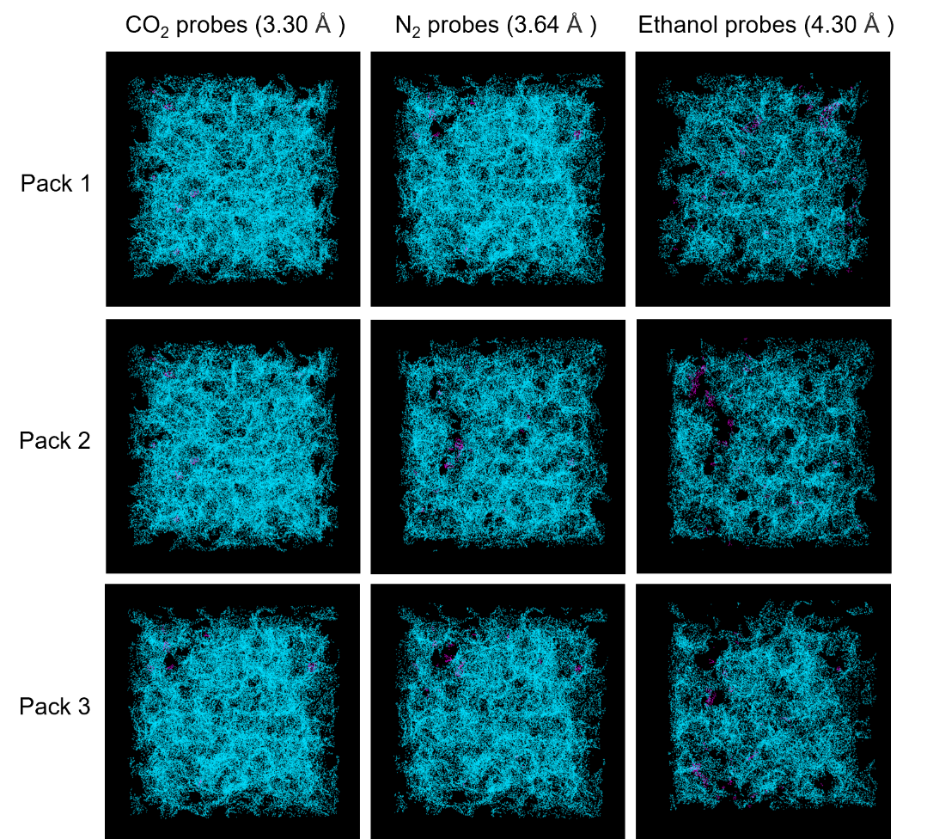


**Figure S16.** Calculated accessible (blue) and non-accessible (magenta) surface area of equilibrated swollen PI-TB-NDI model using three typical probe molecules. Packs 1-3 represent three independent models to adequately sample different structures.

**
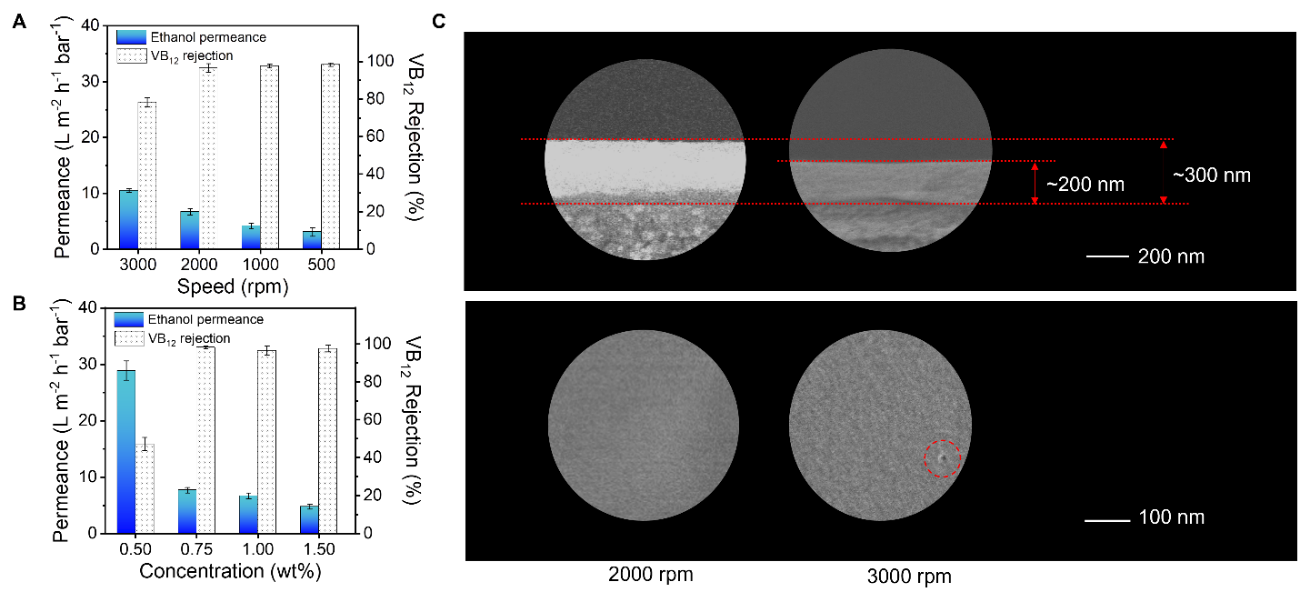
**

**Figure S17.** Ethanol permeation flux and VB_12_ rejection for the TFC membranes prepared with different spin-coating concentrations (spin speed of 2000 rpm) and different spin speeds (concentration of 1.0 wt%). a) Different spin-coating concentrations (spin speed of 2000 rpm). b) Different spin speeds (concentration of 1.0 wt%). The trans-membrane pressure was 0.4 MPa. Ethanol permeance gradually decreased as the spin-coating concentration increased, while the rejection of VB_12_ gradually increased. Similarly, as the spin-coating speed decreased, the ethanol permeance gradually decreased, while the rejection of VB_12_ gradually increased. c) The changes in membrane performance are directly related to the changes in separation layer thickness and defects.


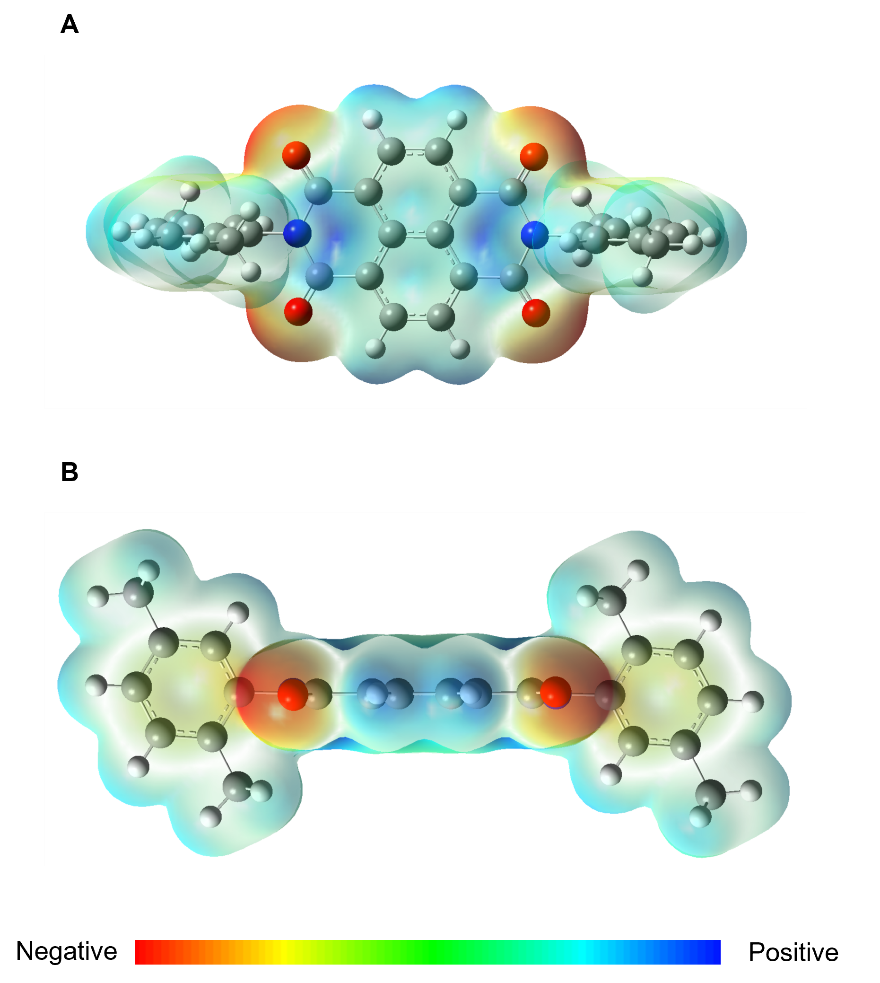


**Figure S18**. DFT-computed electrostatic potential maps (red: electron-rich, blue: electron-poor) calculated by Gaussian 09. a, front view and b, top view.

**Table S1**. Repeating units used in the training and testing set.

| SMILES | Repeat units |
| --- | --- |
| *C(C*)CCCCCCC | 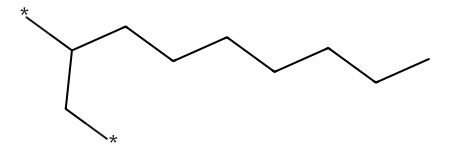 |
| *C(C*)c1c(cccc1)C(=O)Oc1ccccc1 | 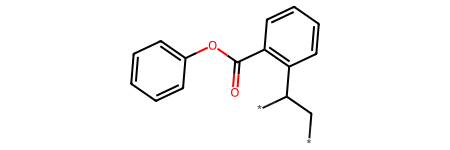 |
| *C(C*)c1ccc(cc1)[Si](C)(C)C | 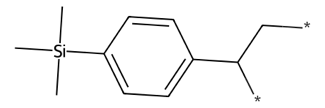 |
| *C(C*)OC(=O)c1cc(ccc1)[N+](=O)[O-] | 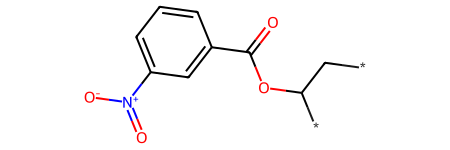 |
| *C(C*)C(=O)OCCCSCCC#N | 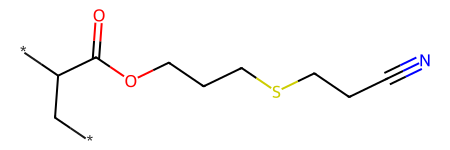 |
| *c1nc2c(nc1c1ccccc1)cc(cc2)c1cc2c(nc(c(n2)c2ccccc2)c2ccc(cc2)Oc2ccc(cc2)*)cc1 | 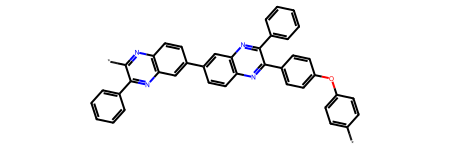 |
| *OC(=O)CCCCC(=O)OCC(C(C(C*)(F)F)(F)F)(F)F | 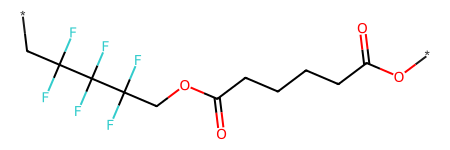 |
| *c1cc2c(C(=O)N(C2=O)c2ccc(cc2)C(=O)c2ccc(cc2)N2C(=O)c3c(C2=O)cc(cc3)C(=O)*)cc1 | 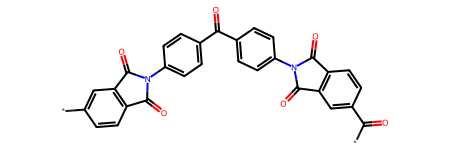 |
| *C(C*)c1c(ccc(c1)C(=O)Oc1ccc(cc1)OCC)C(=O)Oc1ccc(cc1)OCC | 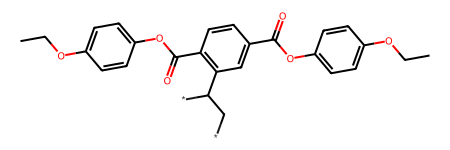 |
| *C(C*)(C)C(=O)OCC1(COC(OC1)CP(=O)(OCC)OCC)COCC1(COC(OC1)CP(=O)(OCC)OCC)COC(=O)C(*)C* | 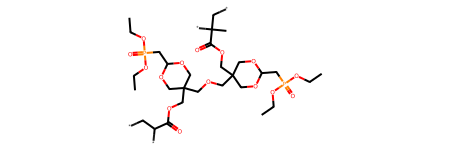 |
| *Oc1ccc(cc1)N=C(c1ccc(cc1)Oc1ccc(cc1)C(c1ccc(cc1)Oc1ccc(cc1)C(=Nc1ccc(cc1)*)c1ccccc1)(C)C)c1ccccc1 | 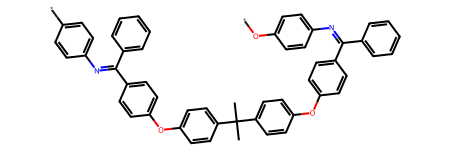 |
| *N1C(=O)c2c(C1=O)cc(cc2)Oc1cc2c(cc1Oc1cc3c(C(=O)N(C3=O)c3ccc(cc3)Oc3c(cccc3)Oc3ccc(cc3)*)cc1)cccc2 | 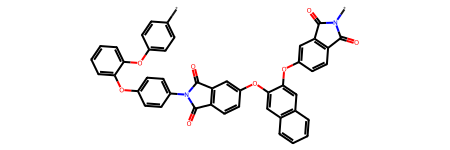 |
| *N1C(=O)c2c(C1=O)cc(cc2)c1cc2c(C(=O)N(C2=O)c2c(cc(cc2)Cc2cc(c(cc2)*)C(C)(C)C)C(C)(C)C)cc1 | 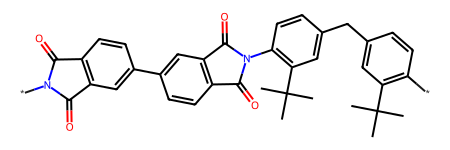 |
| *Nc1cc(ccc1)NC(=O)c1cc(ccc1)C(=O)Nc1ccc(cc1)C(=O)NC(=S)* | 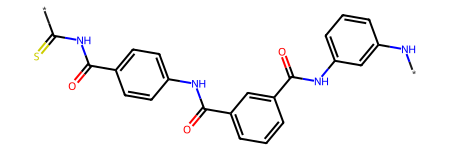 |
| *c1ccc(cc1)C(C(*)(C(=O)OCC(C)C)C#N)(C(=O)OCC(C)C)C#N | 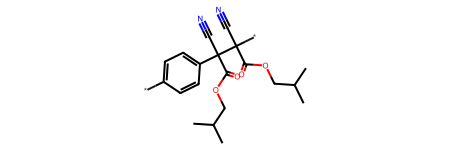 |
| *Nc1ccc(N*)cc1 | 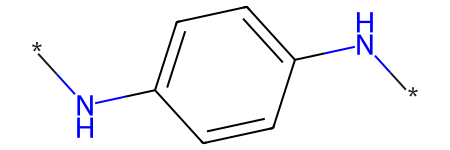 |
| *Nc1c2ccccc2c(N*)c2c(-c3ccccc3)cccc12 | 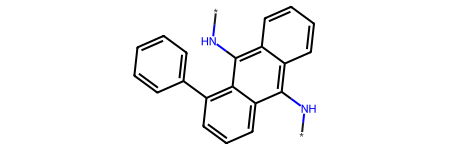 |
| *Nc1ccc([C@@]2(c3ccccc3)c3ccccc3-c3cc(N*)ccc32)cc1 | 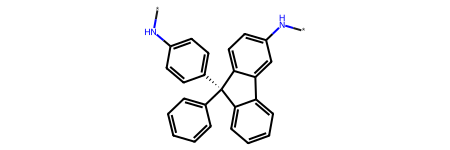 |
| *Nc1ccc2c(c1)C1(CC2)CCc2ccc(N*)cc21 | 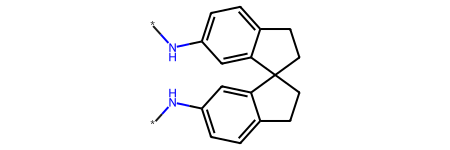 |
| *Nc1ccc([C@]2(C)c3cc(N*)ccc3[C@@H](C)[C@H]2C)cc1 | 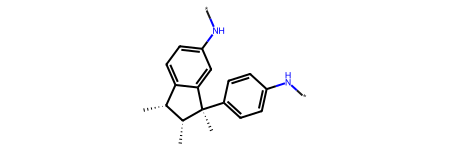 |
| *Nc1ccc2c(c1)C1(c3cc(N)ccc3-2)c2cc(N)ccc2-c2ccc(N*)cc21 | 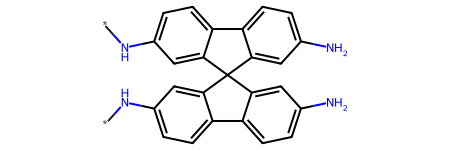 |
| … | … |

**Table. S2.** How these substructures were captured by MFF (Take PIM-1 for example).

| Scan radius | Substructures captured by MFF |
| --- | --- |
| 1 | 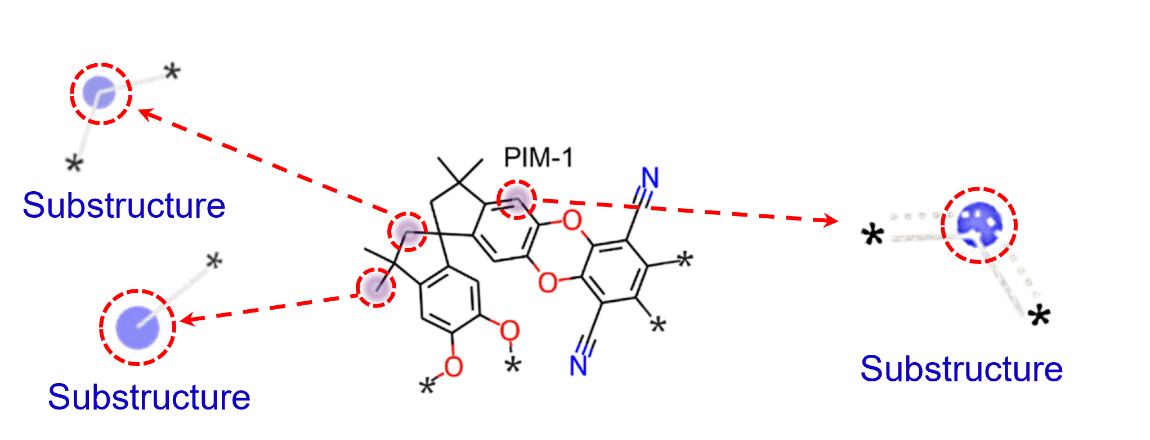 |
| 2 | 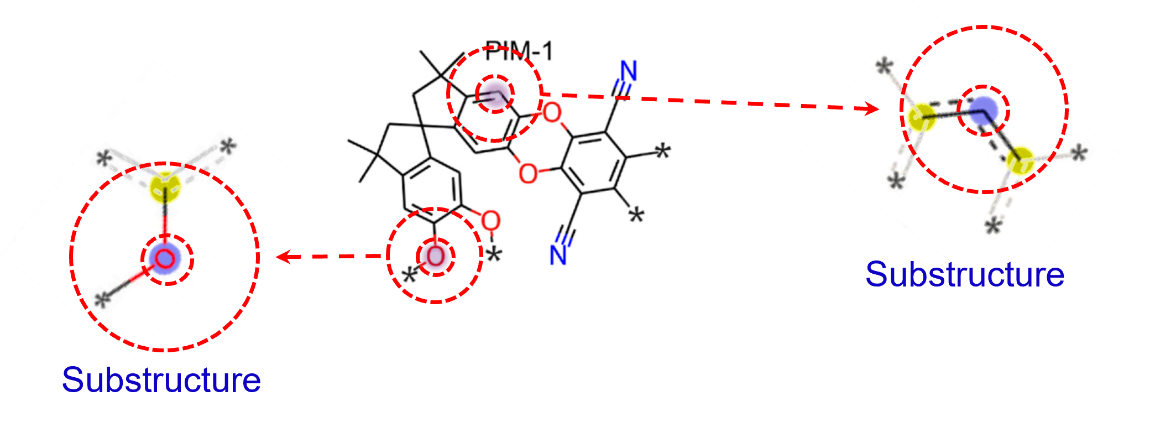 |
| 3 | ... |

* The substructures were captured by the following steps. (1) Molecular structure representation: The polymer structure was represented as a graph, where atoms were represented as nodes and bonds as edges. (2) Scan radius selection: Choosing the scan radius of the MFF. This radius defined the maximum distance to extend from each atom, capturing the neighboring substructures within the fingerprint. (3) Capture of substructures: Starting from each atom, the graph was traversed along paths within the specified radius, recording encountered substructures such as specific atomic environments, bond types, etc. Additionally, “Solid + dashed” lines represented C-C bonds in aromatic rings.

**Table. S3**. Substructures captured by MFF.


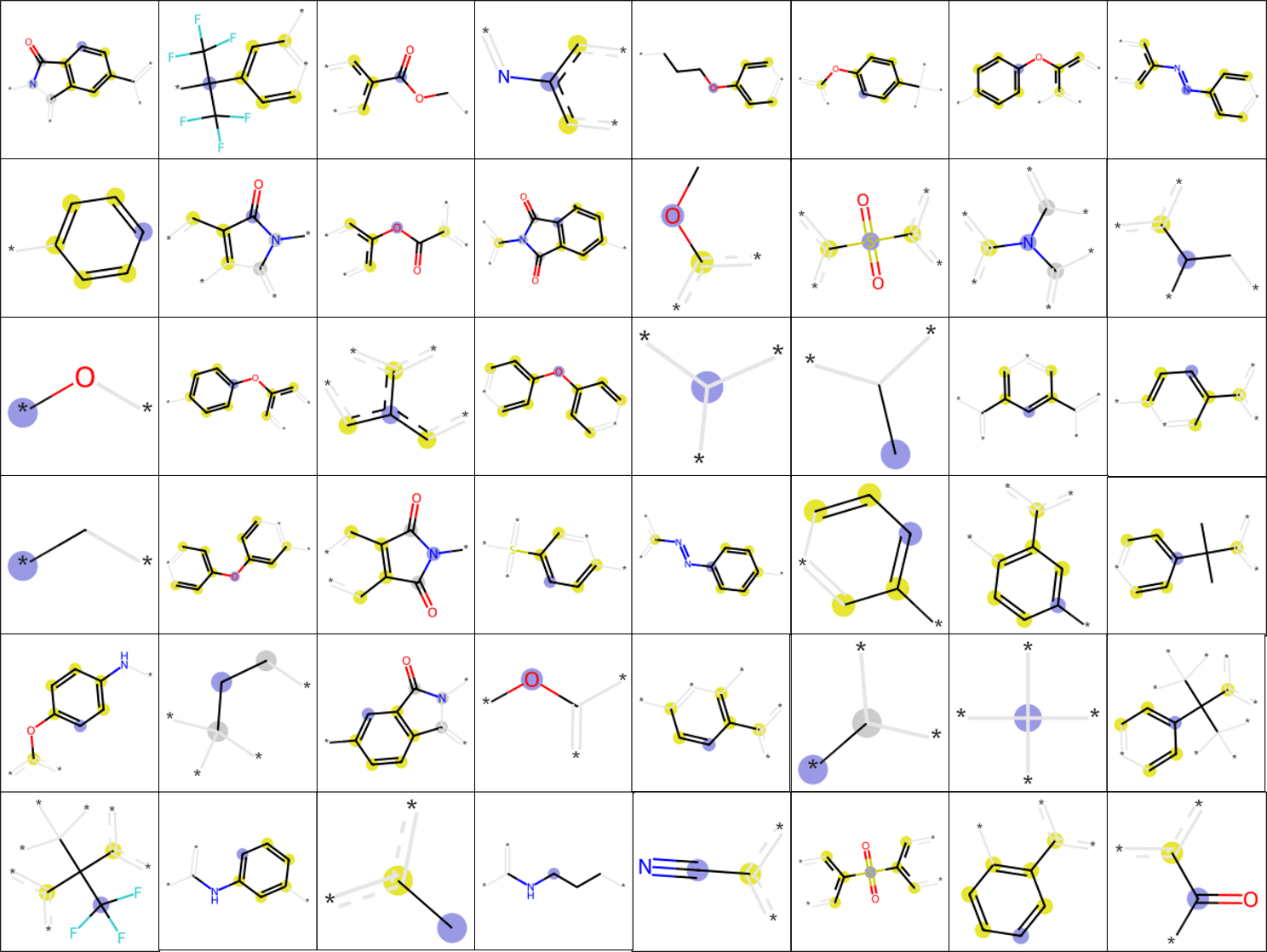


**Table S4**. Predicted fractional free volume (FFV) of PIMs.

| Index | SMILES | Repeat units | Predicted FFV |
| --- | --- | --- | --- |
| 1 | *CN1CN(*)Cc2c(C)cc(C3(c4cc(*)c(*)c(C)c4)c4ccccc4-c4ccccc43)cc21 | 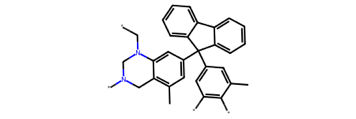 | 0.40990133 |
| 2 | *CN1CN(*)Cc2c1ccc(N1C(=O)c3cc(C)c(C(c4cc5c(cc4C)C(=O)N(c4ccc(*)c(*)c4C)C5=O)c4c(C)cc(C)cc4C)cc3C1=O)c2C | 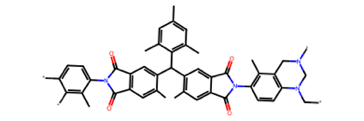 | 0.40449288 |
| 3 | *CN1CN(*)Cc2c1ccc(N1C(=O)c3cc(C)c(C4(c5cc6c(cc5C)C(=O)N(c5ccc(*)c(*)c5C)C6=O)c5ccccc5-c5ccccc54)cc3C1=O)c2C | 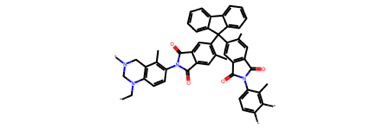 | 0.40193687 |
| 4  This work | *CN1CN(*)Cc2c(C)c(N3C(=O)c4ccc5c6c(ccc(c46)C3=O)C(=O)N(c3cc(C)c(*)c(*)c3C)C5=O)cc(C)c21 | 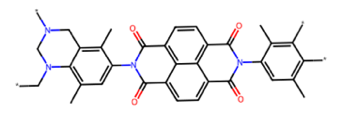 | 0.40128825 |
| 5 | *CN1CN(*)Cc2c(C)c(N3C(=O)c4cccc(-c5ccc6c(c5)C(=O)N(c5cc(C)c(*)c(*)c5C)C6=O)c4C3=O)cc(C)c21 | 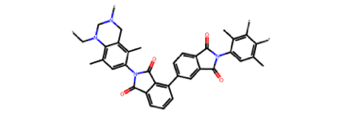 | 0.39868085 |
| 6 | *CN1CN(*)Cc2c(C)c(N3C(=O)c4ccc(C(c5ccc6c(c5)C(=O)N(c5cc(C)c(*)c(*)c5C)C6=O)(C(F)(F)F)C(F)(F)F)cc4C3=O)cc(C)c21 | 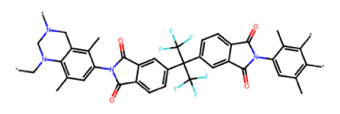 | 0.39807936 |
| 7 | *CN1CN(*)Cc2cc3c(cc21)C1(c2ccccc2-c2ccccc21)c1cc(*)c(*)cc1-3 | 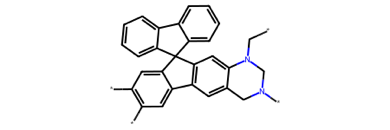 | 0.39766268 |
| 8 | *CN1CN(*)Cc2ccc(C3(c4ccc(*)c(*)c4)c4ccccc4-c4ccccc43)cc21 | 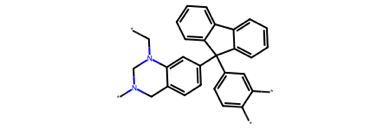 | 0.3961358 |
| 9 | *CN1CN(*)Cc2c(C)c(N3C(=O)c4ccc(C(=O)c5ccc6c(c5)C(=O)N(c5cc(C)c(*)c(*)c5C)C6=O)cc4C3=O)cc(C)c21 | 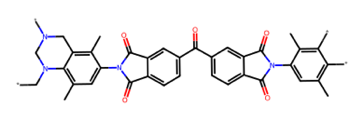 | 0.39379404 |
| 10 | *CN1CN(*)Cc2c1ccc(N1C(=O)c3ccc(-c4ccc5c(c4)C(=O)N(c4ccc(*)c(*)c4C)C5=O)cc3C1=O)c2C | 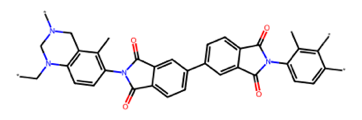 | 0.39340035 |
| 11 | *CN1CN(*)Cc2c(C)c(N3C(=O)C4C5C(=O)N(c6cc(C)c(*)c(*)c6C)C(=O)C5C4C3=O)cc(C)c21 | 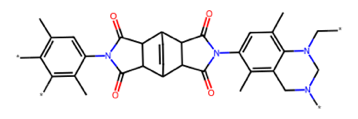 | 0.39261363 |
| 12 | *CN1CN(*)Cc2c(C)c(N3C(=O)c4cccc(Oc5ccc6c(c5)C(=O)N(c5cc(C)c(*)c(*)c5C)C6=O)c4C3=O)cc(C)c21 | 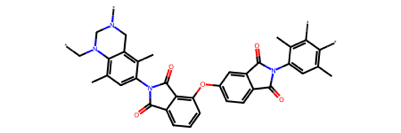 | 0.39201274 |
| 13 | *CN1CN(*)Cc2cc(N3C(=O)c4ccc5c6c(ccc(c46)C3=O)C(=O)N(c3cc(C)c(*)c(*)c3)C5=O)cc(C)c21 | 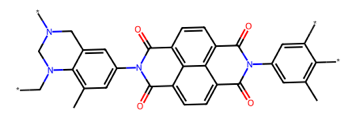 | 0.39200009 |
| 14 | *CN1CN(*)Cc2c(C)c(N3C(=O)c4ccc(Oc5ccc6c(c5)C(=O)N(c5cc(C)c(*)c(*)c5C)C6=O)cc4C3=O)cc(C)c21 | 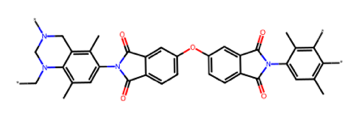 | 0.39147159 |
| 15 | *CN1CN(*)Cc2c(C)c(N3C(=O)C4C5C=CC(C6C(=O)N(c7cc(C)c(*)c(*)c7C)C(=O)C56)C4C3=O)cc(C)c21 | 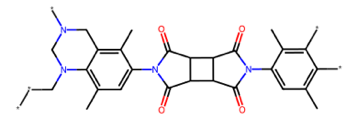 | 0.3914366 |
| 16 | *CN1CN(*)Cc2c1ccc(N1C(=O)c3ccc(C(c4ccc5c(c4)C(=O)N(c4ccc(*)c(*)c4C)C5=O)(C(F)(F)F)C(F)(F)F)cc3C1=O)c2C | 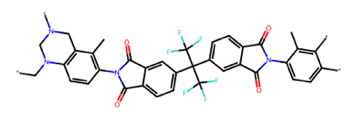 | 0.3901486 |
| 17 | *CN1CN(*)Cc2c1ccc(N1C(=O)c3cccc(-c4ccc5c(c4)C(=O)N(c4ccc(*)c(*)c4C)C5=O)c3C1=O)c2C | 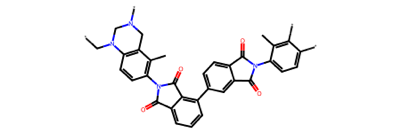 | 0.39014389 |
| 18 | *CN1CN(*)Cc2c1ccc(N1C(=O)c3ccc(C(=O)c4ccc5c(c4)C(=O)N(c4ccc(*)c(*)c4C)C5=O)cc3C1=O)c2C | 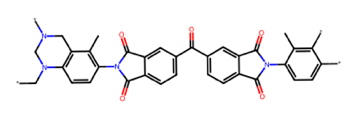 | 0.38597203 |
| 19 | *CN1CN(*)Cc2cc(N3C(=O)c4ccc5c6c(ccc(c46)C3=O)C(=O)N(c3ccc(*)c(*)c3)C5=O)ccc21 | 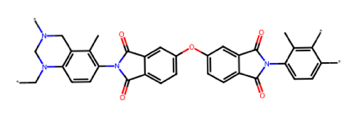 | 0.38546485 |
| 20 | *CN1CN(*)Cc2c1ccc(N1C(=O)c3ccc(Oc4ccc5c(c4)C(=O)N(c4ccc(*)c(*)c4C)C5=O)cc3C1=O)c2C | 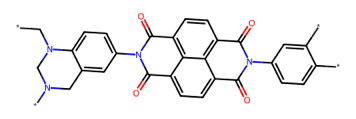 | 0.38364988 |
| 21 | *CN1CN(*)Cc2c1ccc(N1C(=O)c3cccc(Oc4ccc5c(c4)C(=O)N(c4ccc(*)c(*)c4C)C5=O)c3C1=O)c2C | 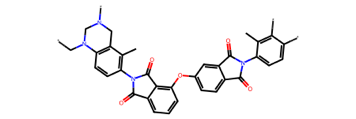 | 0.38346246 |

**Table S5**. Details of solution property and solubility parameters of solvents and PI-TB-NDI taken from different publications.^[27–29]^

| Name | Viscosity  at 25 °C (×10^-3^ Pa s) | Hansen solubility parameter (MPa^1/2^) | | | | Kinetic diameter  (nm) |
| --- | --- | --- | --- | --- | --- | --- |
|  |  | δ_d_ | δ_p_ | δ_h_ | δ_tot_ |  |
| DMSO | 2.00 | 18.4 | 16.4 | 10.2 | 26.7 | 0.49^a)^ |
| Ethanol | 1.08 | 15.8 | 8.8 | 19.4 | 26.5 | 0.43 |
| THF | 0.55 | 16.8 | 5.7 | 5.7 | 19.4 | 0.49 |
| Ethyl Acetate | 0.46 | 15.8 | 5.3 | 7.2 | 18.1 | 0.52 |
| Methanol | 0.60 | 15.1 | 12.3 | 22.3 | 29.6 | 0.38 |
| n-Heptane | 0.41 | 15.3 | 0 | 0 | 15.3 | 0.43 |
| Acetone | 0.33 | 15.5 | 10.4 | 7.0 | 19.9 | 0.47 |
| PI-TB-NDI | NA | 20.9 | 5.4 | 8.2 | 23.1 | N.a. |

^a)^ The kinetic diameter of DMSO was assessed using the molecular geometries obtained by ab-initio calculations.

**Table S6.** Molecular weight and electric charge of dyes and neutral molecules

| Name | Molecular Weight (g mol^-1^) | Electric Charge |
| --- | --- | --- |
| Isatin | 147.13 | 0 |
| Azure B | 305.83 | + |
| Methyl Orange | 327.33 | - |
| Crystal Violet | 407.98 | + |
| Food Yellow 3 | 452.36 | - |
| Basic Blue 26 | 506.09 | + |
| Hexaphenylbenzene | 534.69 | 0 |
| Amido black 10 | 616.49 | + |
| Vitamin B12 | 1355.38 | 0 |

**Table S7.** Comparison of our membrane with advanced OSN membranes reported in references.

| Membrane types | | Permeance^a)^ | Permeability^b)^ | Thickness^c)^ | MWCO^d)^ | Ref. |
| --- | --- | --- | --- | --- | --- | --- |
| TFC-PIM | PI-TB-NDI/XP84 | 7.65 | 1990 | 260 | 500-600 | *This work* |
|  | PIM-1/XP84 | 2.3 | 550 | 237 | 500-800 | ^[30]^ |
|  | PIM-1/PAN | 3 | 900 | 300 | ＞535 | ^[31]^ |
|  | TPIM/XP84 | 1.6 | 250 | 150 | 627 | ^[32]^ |
|  | AOPIM-1/PAN | 15.5 | 1364 | 88 | 800 | ^[33]^ |
|  | PIM−COCl/PAN | 1.8 | 360 | 200 | 408 | ^[34]^ |
|  | PIM-EA-TB/PAN | 2.49 | 1604 | 644 | 1014 | ^[35]^ |
| ISA | Kevlar nanofiber membrane | 2.9 | 232 | 80 | 700 | ^[36]^ |
|  | Crosslinked PI | 1.1 | 303 | 273 | 327 | ^[37]^ |
| TFC-PA | *β-*CD-TMC/Matrimid | 3.8 | 456 | 120 | 400 | ^[38]^ |
|  | PA-MPDTrip/PAN | 2.4 | 813.6 | 339 | 200 | ^[39]^ |
|  | (PI-PI)xa | 2.0 | 840 | 420 | 479 | ^[40]^ |
| TFC-CMP | m-CMP/PAN | 5.5 | 220 | 40 | 560 | ^[41]^ |
| COF membrane | TFP-DHF | 40.0 | 2440 | 61 | 900 | ^[42]^ |

^a)^ Ethanol permeance (L m^-2^ h^-1^ bar^-1^)

^b)^ Ethanol permeability (L m^-2^ h^-1^ bar^-1^ nm)

^c)^ Thickness of separation layer (nm)

^d)^ Molecular weight cut-off (Da)

**References**

[1] L. Tao, J. He, T. Arbaugh, J. R. McCutcheon, Y. Li, *Journal of Membrane Science* **2023**, *665*, 121131.

[2] S. Otsuka, I. Kuwajima, J. Hosoya, Y. Xu, M. Yamazaki, in *2011 International Conference on Emerging Intelligent Data and Web Technologies*, IEEE, Tirana, Albania, **2011**, pp. 22–29.

[3] J. Yang, L. Tao, J. He, J. R. McCutcheon, Y. Li, *Sci. Adv.* **2022**, *8*, eabn9545.

[4] G. Landrum, *Greg Landrum* **2013**, *8*, 31.

[5] L. Breiman, *Machine learning* **2001**, *45*, 5–32.

[6] S. M. Lundberg, S.-I. Lee, *Advances in neural information processing systems* **2017**, *30*.

[7] A. Frisch, *Wallingford, USA, 25p* **2009**, *470*.

[8] T. Lu, F. Chen, *Journal of Computational Chemistry* **2012**, *33*, 580–592.

[9] M. Meunier, S. Robertson, *Molecular Simulation* **2021**, *47*, 537–539.

[10] Paul. J. Flory, M. Volkenstein, *Biopolymers* **1969**, *8*, 699–700.

[11] D. Van Der Spoel, E. Lindahl, B. Hess, G. Groenhof, A. E. Mark, H. J. C. Berendsen, *Journal of Computational Chemistry* **2005**, *26*, 1701–1718.

[12] J. Wang, R. M. Wolf, J. W. Caldwell, P. A. Kollman, D. A. Case, *Journal of computational chemistry* **2004**, *25*, 1157–1174.

[13] L. TIAN, *Sobtop, Version 1.0*, **n.d.**

[14] C. Yang, R. Xu, S. Tang, Y. Zhuang, L. Luo, X. Liu, *Adv. Theory Simul.* **2021**, *4*, 2100016.

[15] M. Haranczyk, C. H. Rycroft, R. L. Martin, T. F. Willems, *Lawrence Berkeley National Laboratoy, Berkeley, 2012á SearcháPubMed* **2012**.

[16] K. A. Thompson, R. Mathias, D. Kim, J. Kim, N. Rangnekar, J. R. Johnson, S. J. Hoy, I. Bechis, A. Tarzia, K. E. Jelfs, B. A. McCool, A. G. Livingston, R. P. Lively, M. G. Finn, *Science* **2020**, *369*, 310–315.

[17] S. Grimme, *J. Comput. Chem.* **2006**, *27*, 1787–1799.

[18] P. Gorgojo, S. Karan, H. C. Wong, M. F. Jimenez-Solomon, J. T. Cabral, A. G. Livingston, *Advanced Functional Materials* **2014**, *24*, 4729–4737.

[19] R. B. Bird, W. E. Stewart, E. N. Lightfoot, *Transport Phenomena 2nd Edition,(2002)*, John Wiley & Sons, **n.d.**

[20] Y. Zhuang, R. Orita, E. Fujiwara, Y. Zhang, S. Ando, *Macromolecules* **2019**, *52*, 3813–3824.

[21] Y. Zhang, W. H. Lee, J. G. Seong, J. Dai, S. Feng, Y. Wan, Y. M. Lee, Y. Zhuang, *Polymer* **2022**, *239*, 124412.

[22] Y. Zhuang, S. Ando, *Polymer* **2017**, *123*, 39–48.

[23] X. Ma, M. Abdulhamid, X. Miao, I. Pinnau, *Macromolecules* **2017**, *50*, 9569–9576.

[24] Y. Zhuang, J. G. Seong, Y. S. Do, W. H. Lee, M. J. Lee, M. D. Guiver, Y. M. Lee, *Journal of Membrane Science* **2016**, *504*, 55–65.

[25] Y. Zhuang, J. G. Seong, Y. S. Do, W. H. Lee, M. J. Lee, Z. Cui, A. E. Lozano, M. D. Guiver, Y. M. Lee, *Chem. Commun.* **2016**, *52*, 3817–3820.

[26] X. Hu, W. H. Lee, J. Zhao, J. Y. Bae, J. S. Kim, Z. Wang, J. Yan, Y. Zhuang, Y. M. Lee, *Journal of Membrane Science* **2020**, *610*, 118255.

[27] I. Smallwood, *Handbook of Organic Solvent Properties*, Butterworth-Heinemann, **2012**.

[28] A. F. M. Barton, *CRC Handbook of Solubility Parameters and Other Cohesion Parameters: Second Edition*, Routledge, New York, **2017**.

[29] M. E. van Leeuwen, *Fluid Phase Equilibria* **1994**, *99*, 1–18.

[30] J. Li, W. Feng, M. Zhang, X. Wang, C. Fang, J. Wang, L. Zhang, L. Zhu, *Macromol. Rapid Commun.* **2023**, *44*, 2200826.

[31] D. Fritsch, P. Merten, K. Heinrich, M. Lazar, M. Priske, *Journal of Membrane Science* **2012**, *401–402*, 222–231.

[32] J. Gao, S. Japip, T.-S. Chung, *Chemical Engineering Journal* **2018**, *353*, 689–698.

[33] Y. Jin, Q. Song, N. Xie, W. Zheng, J. Wang, J. Zhu, Y. Zhang, *Journal of Membrane Science* **2021**, *632*, 119375.

[34] S. Zhou, Y. Zhao, J. Zheng, S. Zhang, *Journal of Membrane Science* **2019**, *591*, 117347.

[35] H. Zhou, A. Akram, A. J. C. Semiao, R. Malpass-Evans, C. H. Lau, N. B. McKeown, W. Zhang, *Journal of Membrane Science* **2022**, *644*, 120172.

[36] S. Yuan, J. Swartenbroekx, Y. Li, J. Zhu, F. Ceyssens, R. Zhang, A. Volodine, J. Li, P. Van Puyvelde, B. Van der Bruggen, *Journal of Membrane Science* **2019**, *573*, 612–620.

[37] H. Mariën, I. F. J. Vankelecom, *Journal of Membrane Science* **2017**, *541*, 205–213.

[38] J. Liu, D. Hua, Y. Zhang, S. Japip, T.-S. Chung, *Advanced Materials* **2018**, *30*, 1705933.

[39] Z. Ali, B. S. Ghanem, Y. Wang, F. Pacheco, W. Ogieglo, H. Vovusha, G. Genduso, U. Schwingenschlögl, Y. Han, I. Pinnau, *Advanced Materials* **2020**, *32*, 2001132.

[40] C. Li, S. Li, L. Lv, B. Su, M. Z. Hu, *Journal of Membrane Science* **2018**, *564*, 10–21.

[41] B. Liang, H. Wang, X. Shi, B. Shen, X. He, Z. A. Ghazi, N. A. Khan, H. Sin, A. M. Khattak, L. Li, Z. Tang, *Nature Chem* **2018**, *10*, 961–967.

[42] D. B. Shinde, G. Sheng, X. Li, M. Ostwal, A.-H. Emwas, K.-W. Huang, Z. Lai, *J. Am. Chem. Soc.* **2018**, *140*, 14342–14349.

**Author Contributions**

**Shuang Guo:** Conceptualization, Methodology, Investigation, Software, Validation, Formal analysis, Writing - Original Draft, Visualization. **Chuanjie Fang:** Supervision, Conceptualization, Validation, Writing - Review & Editing, Visualization. **Jieting Xu:** Software, Methodology. **Jiaqi Li:** Software, Methodology. **Xiaohe Wang:** Conceptualization, Methodology, Software. **Weilin Feng:** Investigation. **Hukang Guo:** Investigation. **Ming Xie:** Conceptualization, Writing - Review & Editing. **Yongbing Zhuang**: Conceptualization, Methodology. **Young Moo Lee:** Conceptualization, Methodology, Writing - Review & Editing. **Liping Zhu:** Supervision, Conceptualization, Funding acquisition, Project administration.
